# Maturity detection and counting of blueberries in real orchards using a novel STF-YOLO model integrated with ByteTrack algorithm

**DOI:** 10.3389/fpls.2025.1682024

**Published:** 2025-11-27

**Authors:** Na Wu, Jie Wu, Zhechen Wang, Yun Zhao, Xing Xu, Yali Wang, Petr Skobelev, Yanan Mi

**Affiliations:** 1 School of Artificial Intelligence and Information Engineering, Zhejiang University of Science and Technology, Hangzhou, China; 2 Department of Zhejiang Hospital, Hangzhou, China; 3 Samara Federal Research Scientific Center, Russian Academy of Sciences, Samara, Russia; 4 Pegasor Oy, Tampere, Finland

**Keywords:** fruit detection, fruit counting, target detection, YOLO, blueberry

## Abstract

**Introduction:**

Blueberries are highly prized for their nutritional value and economic importance. However, their small size, dense clustering, and brief ripening period make them difficult to harvest efficiently. Manual picking is costly and error-prone, so there is an urgent need for automated, high-precision solutions in real orchards.

**Methods:**

We proposed an integrated framework that combined the STF-YOLO model with the ByteTrack algorithm to detect blueberry maturity and perform counting. Together with ByteTrack, it provided consistent fruit counts in video streams. STF-YOLO replaced the YOLOv8 C2f block with a Detail Situational Awareness Attention (DSAA) module to enable more precise discrimination of maturity. It also incorporated an Adaptive Edge Fusion (AEF) neck to enhance edge cues under leaf occlusion and a Multi-scale Neck Structure (MNS) to aggregate richer context. Additionally, it adopted a Shared Differential Convolution Head (SDCH) to reduce parameters while preserving accuracy.

**Results:**

On our orchard dataset, the model achieved an mAP50 of 79.7%, representing a 3.5% improvement over YOLOv8. When combined with ByteTrack, it attained an average counting accuracy of 72.49% across blue, purple, and green maturity classes in video sequences. Cross-dataset tests further confirmed its robustness. On the MegaFruit benchmark (close-range images), STF-YOLO achieved the highest mAP50 for peaches (91.6%), strawberries (70.5%), and blueberries (90.6%). On the heterogeneous PASCAL VOC2007 dataset, it achieved 66.3% mAP50, outperforming all lightweight YOLO variants across 20 everyday object categories.

**Discussion:**

Overall, these results suggest that the STF-YOLO integrated with the ByteTrack framework can accurately detect and count blueberries in orchards. This lays a solid foundation for the future development of automated blueberry harvesting machinery and improvements in harvest efficiency.

## 1 Introduction

Blueberry is among the world’s most valuable fruit crops from an economic perspective, and is popular with consumers due to their high antioxidant, vitamin and mineral content, which provides anti-inflammatory and anti-cancer benefits (Cheng et al., 2024). However, despite surging demand and cultivation, harvesting remains the bottleneck of the supply chain in China, where picking depends almost entirely on manual labor. In practice, two issues are critical. Firstly, labor shortages during the peak season mean that workers must harvest continuously within the narrow 24-72-hour optimum window. Fatigue leads to inconsistent maturity selection and lower picking efficiency. Secondly, field studies reported that manual compression causes 15–20% fruit damage (Ali et al., 2015). These challenges are exacerbated by the delicate texture, dense clustering and short ripening period of blueberries, all of which complicate large-scale daily harvests (Ding et al., 2023). Consequently, the development of automated, high-precision detection and picking systems has become essential for minimizing losses and meeting market demand. However, beyond these obvious challenges, a deeper issue affecting the entire supply chain has long been overlooked: the lack of accurate yield forecasts. Blueberry growers must make critical decisions weeks before the start of the harvest season, including labor recruitment, procurement of packaging materials, arrangements for cold chain logistics, and coordination of sales channels. These decisions rely heavily on estimates of the harvestable fruit yield in kilograms for the next one to two weeks. Currently, such estimates rely almost entirely on farmers' experience, resulting in high levels of subjectivity and significant errors. This frequently leads to the misallocation of resources and economic losses. Therefore, developing technology that can objectively and accurately assess the quantity and distribution of fruit maturity within orchards is fundamental to achieving precision agricultural management and intelligent decision-making. The significance of this technology extends far beyond addressing issues specific to the harvesting process alone.

In recent years, a growing body of research has sought to develop more accurate methods of detecting fruit. Traditional machine learning approaches, such as Support Vector Machines (SVMs), Random Forests and Classification and Regression Trees (CART), have been explored (Breiman, 2001; Breiman et al., 2017). However, they are limited by their reliance on handcrafted features, which often struggle to generalize. This leads to degraded performance when dealing with challenges like partial occlusion, where the model must infer objects from incomplete information, and significant variations in fruit size, color, or maturity. Consequently, researchers have turned to deep learning techniques, which have stronger feature extraction capabilities and enable reliable recognition in complex orchard scenes. For instance, Zhao et al. (2024) proposed RT-DETR-Tomato, a two-stage detector that combines region proposal and refinement steps to deliver precise tomato localization. Among single-stage detectors, the YOLO (You Only Look Once) family offered an excellent balance of speed and accuracy and became the mainstream choice for fruit detection (Redmon, 2016; Redmon and Farhadi, 2017; Farhadi and Redmon, 2018). Zhang et al. (2022) used YOLOv3 to track citrus fruits in videos and eliminate duplicate detections caused by overlap. Zhao et al. (2024) enhanced YOLOv5 with ShuffleNetv2 and CBAM attention (YOLO-Granada) for lightweight yet accurate pomegranate detection, while An et al. (2022) upgraded YOLOX with C3HB, NAM attention and SIOU loss (SDNet) to identify strawberry growth stages. Collectively, these studies demonstrate that single-stage YOLO variants and carefully optimized two-stage detectors can significantly improve the efficiency and accuracy of fruit detection tasks, thereby highlighting their important role in making harvesting workflows more efficient and increasing agricultural productivity.

Maturity is a crucial indicator for determining the optimal harvest time, as harvesting too early or too late can negatively impact flavor, quality, and economic value (Krishna et al., 2023). In recent years, researchers have dedicated efforts to studying advanced methods for fruit maturity detection. For example, Xiao et al. (2023) developed a YOLOv8-based apple maturity detection model that uses transfer learning to enhance feature extraction and a custom dataset for precise classification. Lai et al. (2022) proposed a YOLOv4-based system for real-time identification of mature oil palm fruit clusters, achieving an mAP50 of 87.9%. Yang et al. (2023) introduced LS-YOLOv8s, a strawberry maturity grading model combining YOLOv8s with an LW-Swin Transformer module, which attained 94.4% accuracy. Chen et al. (2024) designed a multi-task loss function using Scale-Invariant IoU (SIoU) to replace CIoU, improving the accuracy of YOLOv7-based DCNNs in detecting tomato clusters and maturity. Similarly, Wang et al. (2024) integrated Variable Focal Loss (VFL) and Wise-IoU (WIoU) into NVW-YOLOv8s for real-time tomato maturity detection and segmentation. Collectively, these studies demonstrate that deep learning models achieve remarkable results in fruit maturity detection, particularly for large fruits or those with simple backgrounds.

Compared to large fruits like apples and potatoes—which exhibit relatively distinct shapes and fewer occlusion issues—small fruits present additional detection challenges due to dense distribution and severe occlusion. Xie et al. (2022) proposed an improved YOLOv5-litchi model integrating a convolutional block attention module and a small-object detection layer, achieving a 12.9% higher mAP50 than the original YOLOv5. Similarly, Yu et al. (2024) developed a lightweight SOD-YOLOv5n model for winter jujube detection, improving mAP50 by 3% while enabling real-time fruit counting. Gai et al. (2021) further optimized YOLOv4 for occluded and overlapping cherry fruits, increasing mAP50 by 15% over the baseline model. However, these successes are often demonstrated on relatively large fruits or in controlled, close-range imaging scenarios.

Blueberries are particularly challenging to detect due to their small size, clustered growth and color similarity to the background, which makes maturity assessment and counting more complex and relative to many other fruits, detection accuracies on blueberries are often lower. MacEachern et al. (2023) applied six YOLO models to detect blueberries at three different maturity stages. They achieved a mAP50 of 79.79%. Liu et al. (2023) proposed an enhanced YOLO-based algorithm for blueberry maturity detection. This integrates a lightweight CBAM (Little-CBAM), an improved MobileNetv3 backbone and a multi-scale fusion module (MSSENet). This boosted the algorithm's ability to detect small targets and its anti-interference capabilities. Adopting EIOU_Loss and optimizing the anchor frames enabled the method to achieve a mAP50 of 78.3%, which was 9% higher than that of YOLOv5x. Most prior studies have relied on close-range imagery. Moreover, because these examples all used close-range blueberry images (typically 20 cm-50 cm above the canopy), they cannot capture the full scene and are therefore not well suited for integration into field-harvesting robots. However, to capture the entire blueberry crop, the person often stood around 1.5 m-2 m away, depending on the length of the branches and buds. This reduced the clarity of the fruit and, in turn, the accuracy of detection. Zhao et al. (2024) acquired blueberry images using a drone at a height of approximately 5 m above the ground and improved the PF-YOLO model by applying location coding and fast convolution technology, which only increased the mAP50 from 48.9% to 54.4%. In summary, while recent advancements in YOLO-based models have achieved high detection accuracy or blueberries in close-range imagery, this paradigm is fundamentally incompatible with practical field applications requiring whole-plant visibility for tasks like accurate counting. As demonstrated by Zhao et al. (2024), detection performance suffers drastically at the medium-to-long range distances necessary to capture the full extent of a blueberry bush within a single frame. This significant accuracy gap underscores a critical challenge: robust blueberry detection and counting in operational field settings inherently demands imagery encompassing the entire plant profile, inevitably captured from distances where fruit clarity is reduced. Consequently, deploying effective harvesting or scouting robots necessitates overcoming the inherent difficulties of small-target detection in complex, wide-field views obtained from these practical stand-off positions, a core focus of this research.

Accurate yield estimation and maturity assessment are crucial prerequisites for the automated harvesting of blueberries, as they directly influence management decisions and efficiency across the entire production chain. However, real orchard scenarios often include fruits at varying maturity levels, with significant variability not only between individual plants but also within fruit clusters, making consistent visual assessment difficult. This complexity is further exacerbated by the berries’ small size, dense clustering, and color similarity to foliage. Currently, maturity assessment relies heavily on subjective empirical estimates or labor-intensive sampling methods, both of which lack the accuracy and scalability required for large-scale, continuous monitoring. Moreover, precision agriculture demands precise, real-time orchard data to optimize harvest schedules, streamline picking routes, and manage post-harvest logistics effectively. Therefore, there is an urgent need for automated, intelligent detection methods that can objectively and accurately quantify blueberry maturity and yield, providing consistent, reproducible, and timely data to reduce reliance on manual labor, minimize fruit loss, and enhance operational efficiency and profitability (Lobos et al., 2014; DeVetter et al., 2022).

This study focuses on developing an improved detection method for assessing and counting blueberry maturity in real orchard environments. The main contributions are as follows:

1. STF-YOLO (Small Target Fruit YOLO): An enhanced YOLO model for blueberry maturity identification.
2. Innovative Architectural Modules: Detail Situational Awareness Attention (DSAA): Dynamically allocates attention weights. Adaptive Edge Fusion (AEF): Enhances contour representation. Multi-Scale Neck Structure (MNS): Improves small-target detection. The original head structure is replaced with a Shared Differential Convolution Head (SDCH), leveraging shared convolutions to reduce model complexity while enhancing performance.
3. Detection-to-Counting Framework: Blueberry fruits are first detected in individual images, then precisely counted using the ByteTrack algorithm. While performance varies across maturity categories, the method demonstrates robust overall performance in addressing practical challenges in complex orchard environments—highlighting its applicability and reliability.

The remainder of this paper is organized as follows: Section 2 details the blueberry dataset construction and model development. Section 3 presents experimental results, Section 4 provides discussion, and Section 5 concludes with findings and future research directions.

## 2 Materials and methods

### 2.1 Data collection

**Figure 1:**
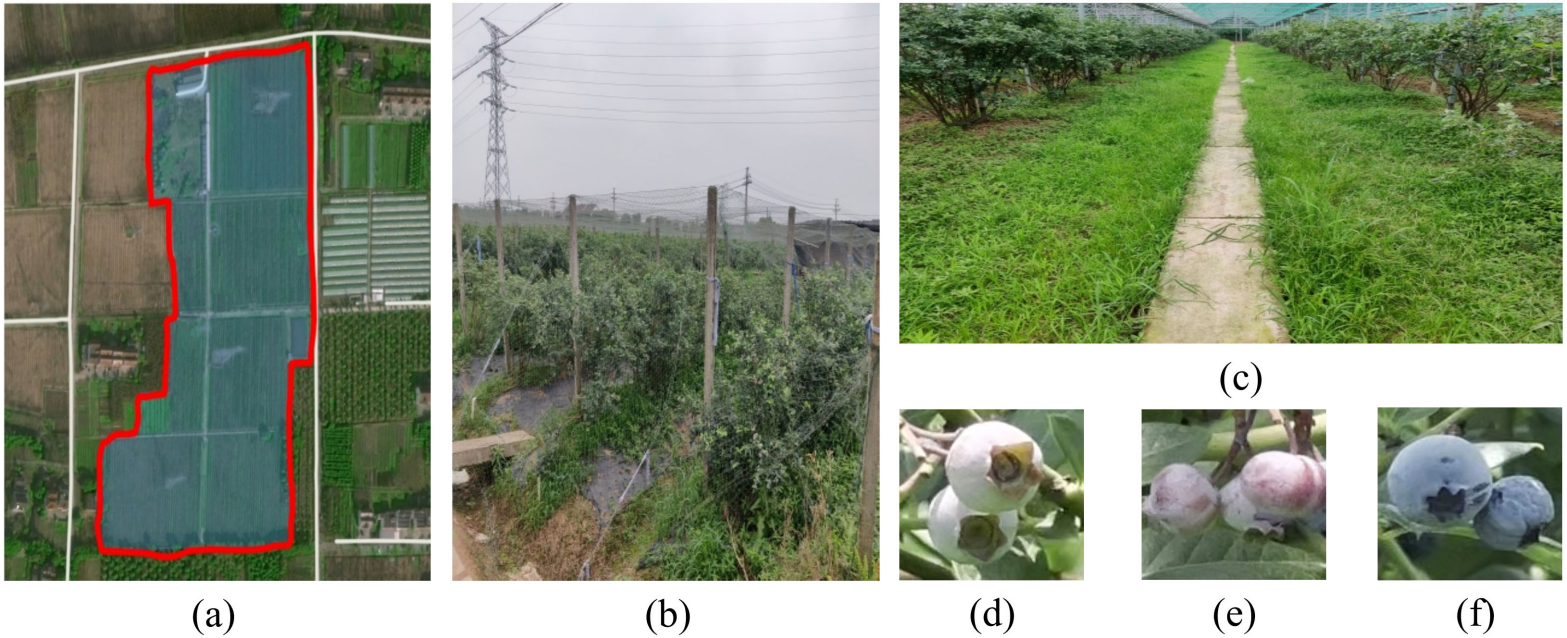
Data collection area, field scenes, blueberry fruit sample images. **(a-c)** Data collection locations and scenarios, **(d-f)** examples of blueberry fruit images at different maturity levels.

We collected high-resolution videos of blueberry plants at Shimen Blueberry Orchard (120°26'44.39" E, 30°39'36.91" N) in Tongxiang City, Jiaxing Prefecture, Zhejiang Province, China, during the ripening period from May to June 2024. Videos were recorded using an iPhone 13 Pro at 3840×2160 pixel resolution between 9:00 AM and 5:00 PM under optimal lighting conditions. The camera was positioned 80–100 cm from the plants to balance detailed feature capture with clarity. During collection, we carefully selected representative blueberries and backgrounds to reflect varying growth conditions across plants. **Figure 1** provides an overview of the data collection process. Panel (a) shows the exact geographic collection area (marked in red). Panels (b) and (c) depict typical field scenes, including planting environments and infrastructure like support structures and pathways. Panels (d), (e), and (f) illustrate blueberries at different maturity stages, highlighting developmental variations in size, color, and appearance.

After recording the videos, we extracted 891 images of blueberry plants, one frame at a time, in order to create a dataset. Extracting specific frames from the videos enabled us to create a comprehensive dataset aligned with practical applications.

### 2.2 Data processing and construction

**Figure 2:**
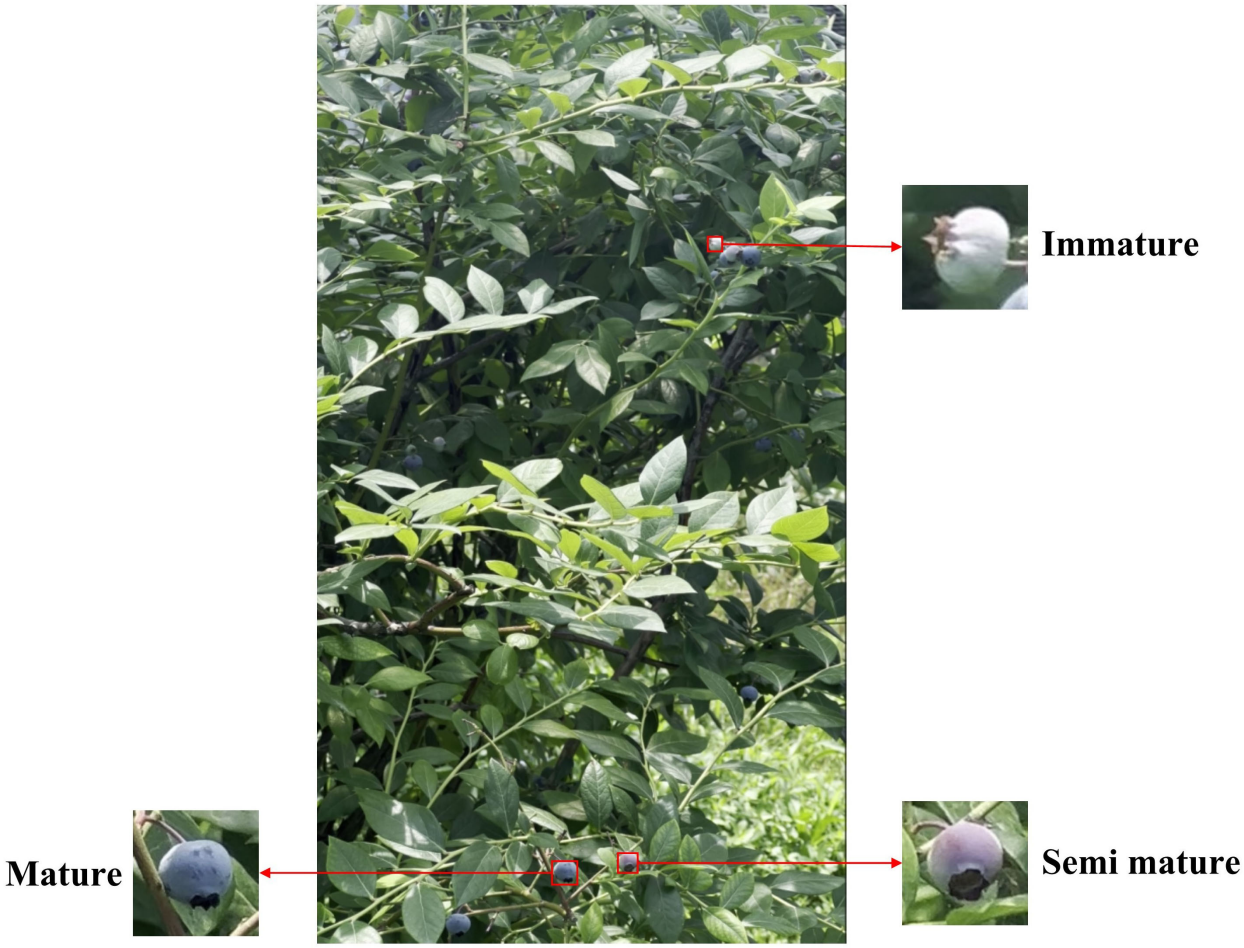
Annotated example.

We manually annotated the aforementioned images using the LabelImg tool (Tzutalin, 2015), as shown in **Figure 2**. The annotation standard was the minimum bounding rectangle surrounding the blueberry fruits, including both complete and partially occluded blueberries.

Blueberries typically grow in clusters, often containing fruits at different maturity stages. Consistent with the method described by Yang et al. (2022), we visually assessed maturity based on color, categorizing fruits as mature (blue), semi-mature (purple), or immature (green). To ensure annotation accuracy, each image was magnified to at least 200% to accommodate the berries' small size. All labels underwent independent review and correction before inclusion in the final dataset. Upon completion, each annotated image corresponded to a TXT file containing category and coordinate information. For fruit detection model development and validation, we randomly allocated 70% of images (623) for training, 15% (134) for validation, and 15% (134) for testing. We applied various data augmentation techniques—including rotation, Gaussian noise addition, flipping, and scaling—to the training and validation sets to enhance training effectiveness and generalization capability. This augmentation yielded 3115 training images and 665 validation images. Finally, to alleviate hardware memory constraints during training, we resized all input images from 3,840 × 2,160 pixels to 640 × 640 pixels. The sample distribution is shown in **Table 1**.

**Table 1:** Dataset sample distribution.

Number of enhanced pictures	Number of train, val, test	Maturity	Labels
3914	Train set of 3115 images	mature	16120
		immature	15420
		semi-mature	7780
	Val set of 665 images	mature	3195
		immature	3025
		semi-mature	1355
	Test set of 134 images	mature	702
		immature	680
		semi-mature	291

### 2.3 Improved YOLOv8 algorithm

#### 2.3.1 YOLOv8 network

**Figure 3:**
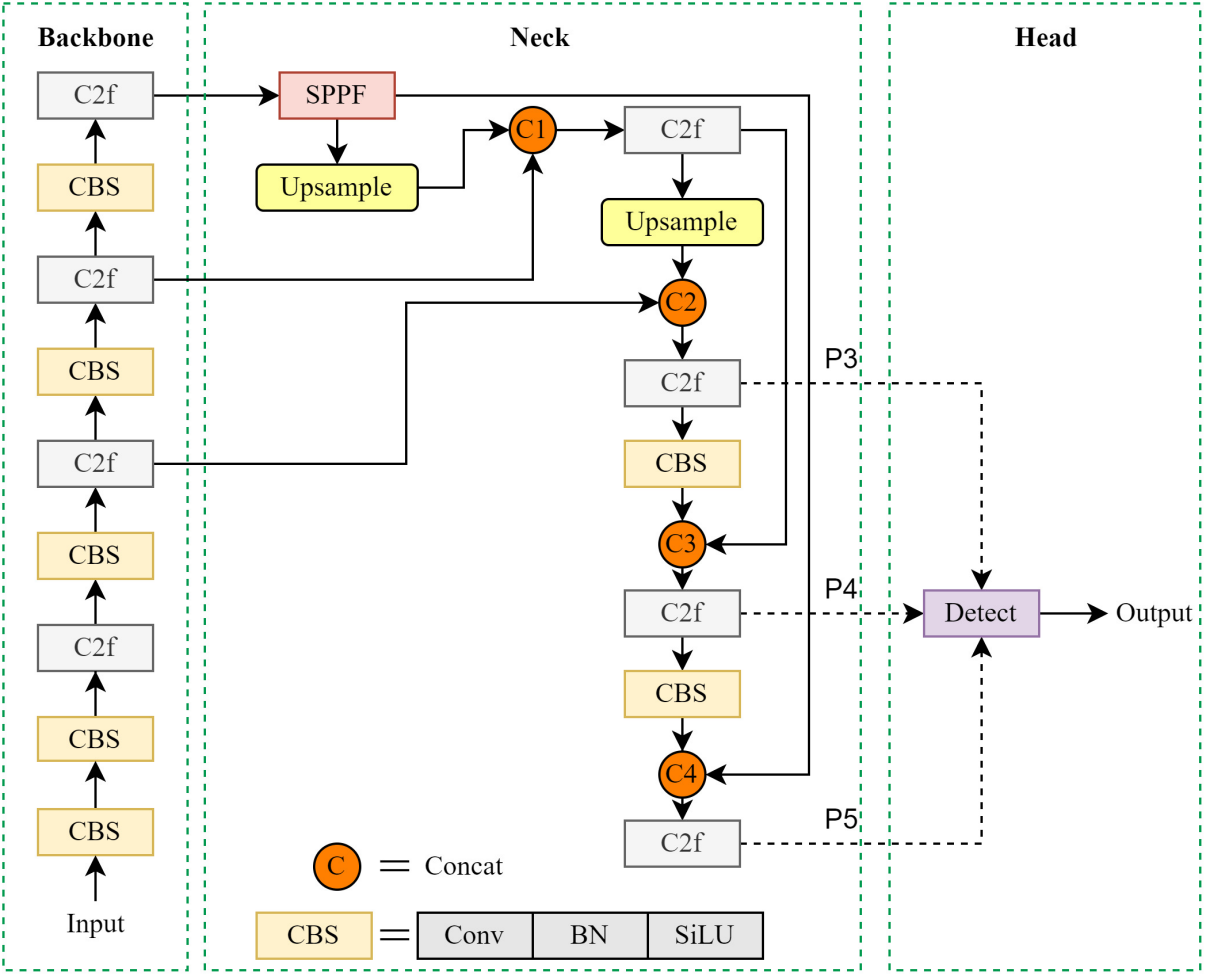
Structure of YOLOv8.

The YOLO series of object detection models is widely acclaimed for its efficient end-to-end detection capabilities and has been extensively used in computer vision tasks including object detection, image segmentation, and target tracking (Wu et al., 2013). YOLOv8, the latest iteration of this series, introduces significant architectural optimizations designed to enhance both accuracy and efficiency in real-time detection (Sohan et al., 2024). Compared to its predecessors (YOLOv5, YOLOv6, YOLOv7), YOLOv8 incorporates a more efficient feature extraction backbone and lightweight structures, reducing computational overhead while improving overall performance. The YOLOv8 architecture comprises three main components, as illustrated in **Figure 3**:

1. Backbone: YOLOv8's backbone integrates advanced modules like ConvNext and Swin Transformer, significantly enhancing feature extraction capabilities. The inclusion of an improved E-ELAN structure and efficient residual modules strengthens its ability to capture contextual information and spatial details. This makes the model particularly suitable for detecting small objects in dense scenes with complex backgrounds.
2. Neck: The neck employs an enhanced feature pyramid network (optimized from PAFPN) for efficient multi-scale feature map fusion. Incorporating spatial and channel attention mechanisms allows the model to adaptively focus on salient feature regions. This improves feature transmission and fusion effectiveness, boosting detection performance for small objects and complex backgrounds.
3. Head: The detection head utilizes dynamic convolution and adaptive feature weighting strategies for precise bounding box and class prediction. An improved positive/negative sample matching algorithm enhances multi-scale detection robustness. The decoupled head structure processes classification and regression tasks separately, mitigating task conflict and further increasing detection accuracy.

Given YOLOv8's outstanding object detection performance, we selected it as the baseline model for high-performance blueberry fruit detection.

#### 2.3.2 STF-YOLO

**Figure 4:**
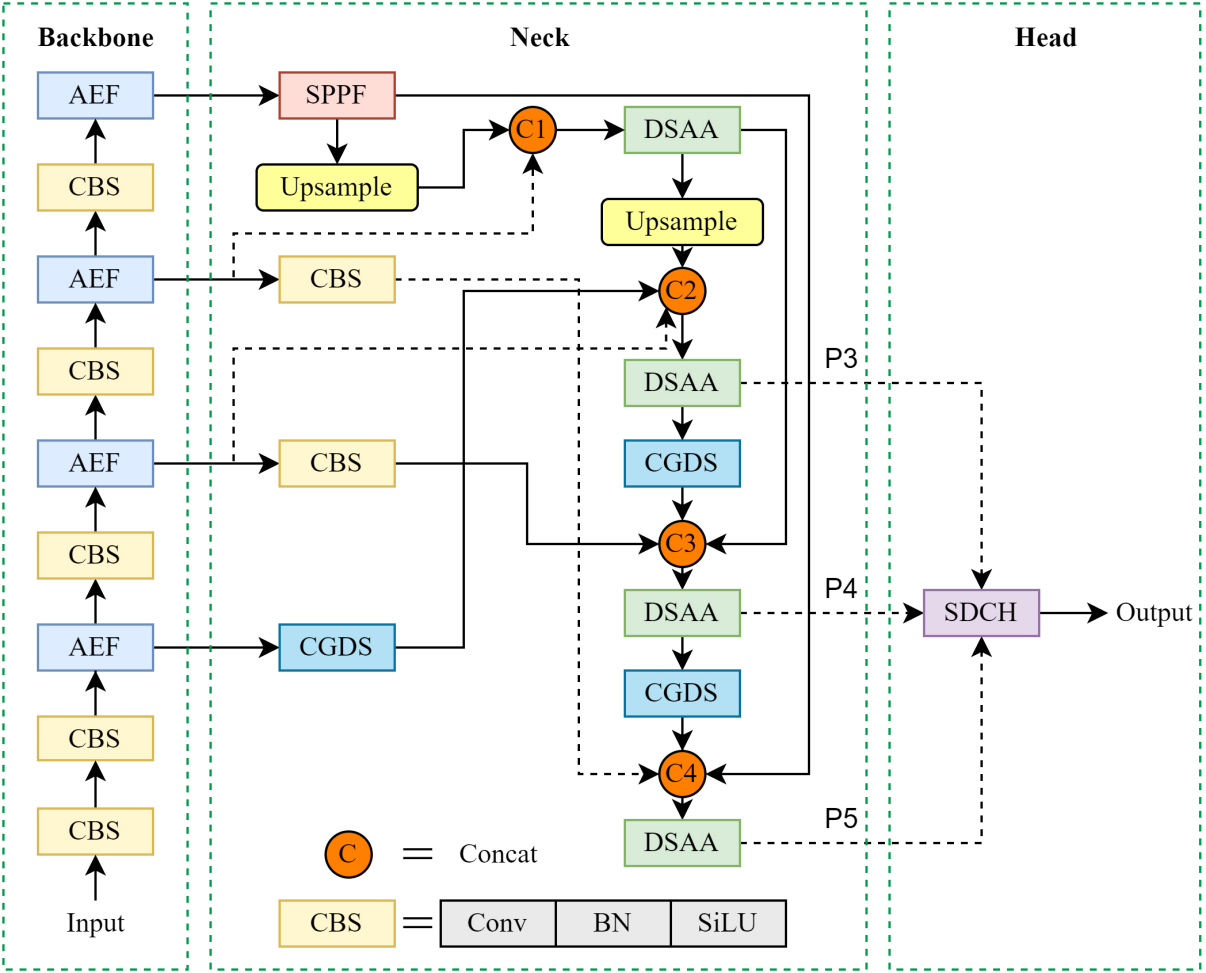
Structure of STF-YOLO.

To address challenges in blueberry fruit detection, including (1) small object sizes, (2) difficulties in assessing maturity, and (3) occlusion caused by overlapping fruits or foliage, we propose an enhanced model STF-YOLO, which is based on YOLOv8. STF-YOLO integrates DSAA, AEF, MNS and SDCH, effectively enhancing both the precision and efficiency of blueberry fruit detection. Its architecture is illustrated in **Figure 4**. The following sections provide a comprehensive explanation of the improvements made to each module.

##### 2.3.2.1 Detail situational awareness attention

**Figure 5:**
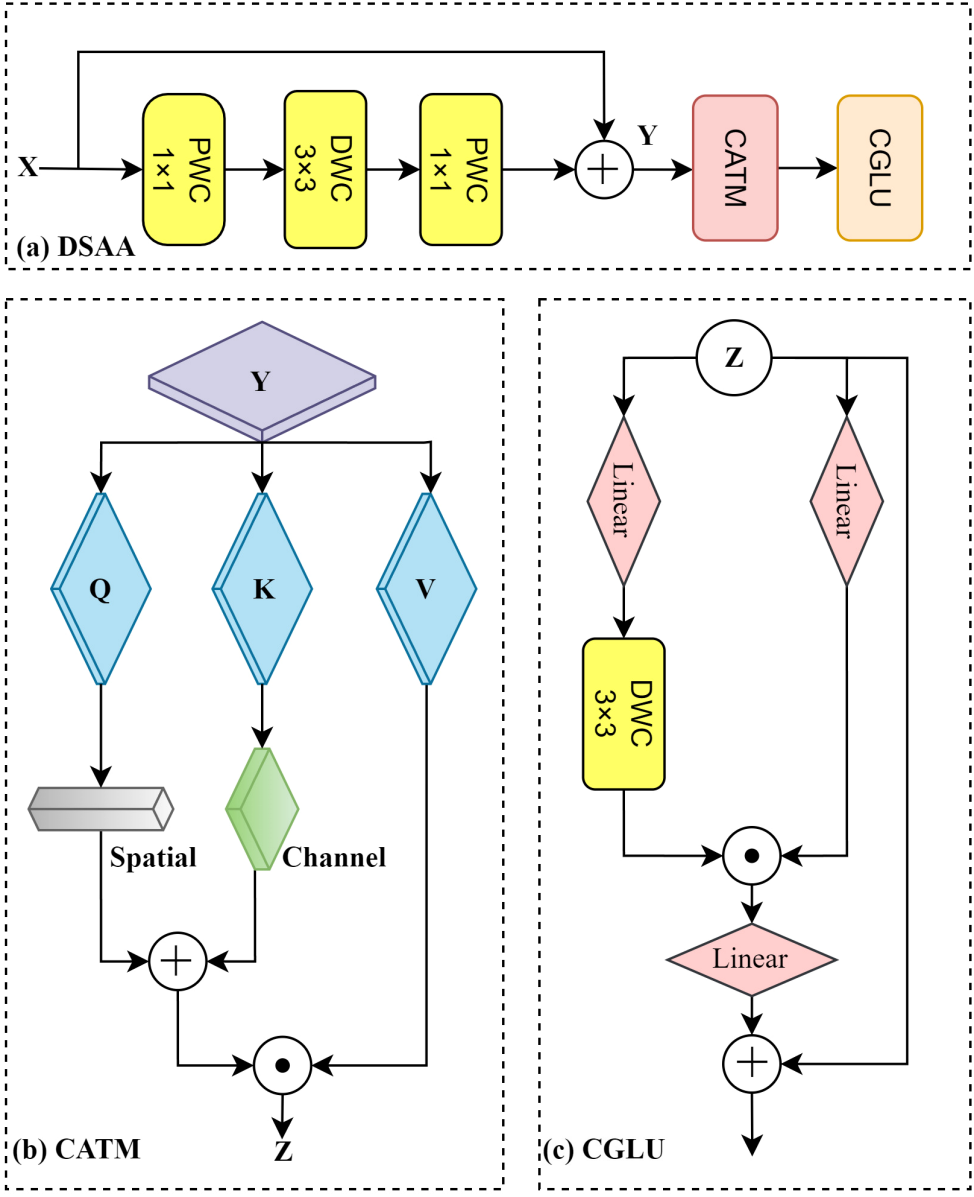
Detail situational awareness attention structure.

In the context of blueberry fruit detection, intricate environmental factors such as lighting and occlusion have been observed to impede the clarity and definition of the fruit's edges and intricate features, thereby exerting a detrimental influence on the model's detection performance. Consequently, the detection model must possess robust detail perception capabilities to accurately distinguish fruits at different maturity levels. The issue under discussion has been addressed by the design of the DSAA module (see **Figure 5a**). This module consists of three main components: basic feature extraction, the Convolutional Additive Token Mixer (CATM) for capturing contextual information, and the Convolutional Gated Linear Unit (CGLU) for enhancing feature selection and improving the model's ability to focus on relevant details.

The C2f structure in YOLOv8 primarily focuses on aggregating overall information during feature extraction but lacks sufficient capability to perceive detailed information. This limitation prevents it from effectively capturing the edge features of blueberry fruits and the fine-grained information related to their maturity. Inspired by Zhang et al. (2024), CATM introduces Query (Q), Key (K), and Value (V) representations after the basic feature extraction to capture contextual information between features. As shown in **Figure 5b**), the module adaptively adjusts Q and K through spatial operations, which use local convolutions (e.g., 3×3 convolutions) to enhance positional relationships, and channel operations, which employ global average pooling and 1×1 convolutions to refine inter-channel dependencies. This results in a weighted attention map as described in Equation 1.

(1) $$\begin{array}{c} Attention(Q,K,V)=Softmax(\frac{QK^{T}}{\sqrt{d_{k}}})V \end{array}$$

where *Q*, *K*, and *V* represent the feature matrices for Query, Key, and Value, respectively. The term $\sqrt{d_{k}}$ denotes the dimension of the Key, used for scaling to prevent excessively large gradients. Spatial operations enhance the spatial positions of the feature maps by emphasizing the relative positional relationships between pixels through local convolutions (e.g., 3×3 convolutions). This allows the model to better capture the detailed edges and shape variations of the fruits, which are critical for identifying subtle differences in blueberry maturity levels, such as size, contour, and texture changes. Channel operations optimize inter-channel dependencies using global average pooling and 1×1 convolutions, enabling the model to focus on the feature channels related to fruit maturity, such as color changes and texture details.

For feature fusion, CATM combines the spatially and channel-wise weighted *Q* and *K* by adding them together and then multiplying by *V*, resulting in the final attention-weighted features. This design effectively captures the spatial relationships of blueberry fruits and identifies specific detail patterns across different channels, which are crucial for assessing fruit maturity.

As shown in **Figure 5c**), the feature maps are forwarded to the CGLU module for further processing to enhance feature discrimination (Shi, 2024). The core idea of CGLU is to regulate the feature flow through a gating mechanism, thereby enhancing the focus on important features while maintaining a lightweight structure. The basic operation of CGLU is illustrated in Equation 2:

(2) $$\begin{array}{c} Gated\;Feature=\;\sigma (W_{1}*X+b_{1})⊙X \end{array}$$

Where *σ* represents the Sigmoid activation function, which is used to generate gating signals; *X* denotes the input feature map; $W_{1}$ and $b_{1}$ are the weights and biases of the convolutional layer, respectively; *∗* signifies the convolution operation; and *⊙* represents element-wise multiplication (Hadamard Product). The gating mechanism dynamically adjusts the feature flow by weighting the input feature map *X* and outputting the weighted feature map, thereby enabling dynamic modulation of the feature stream. Through this approach, CGLU enhances the focus on useful features while suppressing interference from irrelevant ones, further improving the model's ability to distinguish blueberry maturity levels.

The DSAA module replaces the traditional C2f structure by combining basic feature extraction with advanced mechanisms to enhance feature representation. This integration significantly improves the model's ability to detect subtle features, such as fruit edges and maturity-related patterns, while enabling it to handle complex scenarios like varying lighting, angles, and occlusions, ensuring robust and reliable performance in real-world applications.

##### 2.3.2.2 Adaptive edge fusion

In the task of blueberry fruit maturity detection, occlusions often result in blurred or incomplete edge information of the fruits, posing challenges for the model in accurately identifying the fruits and assessing their maturity levels. To address this issue, we propose an AEF module.

**Figure 6:**
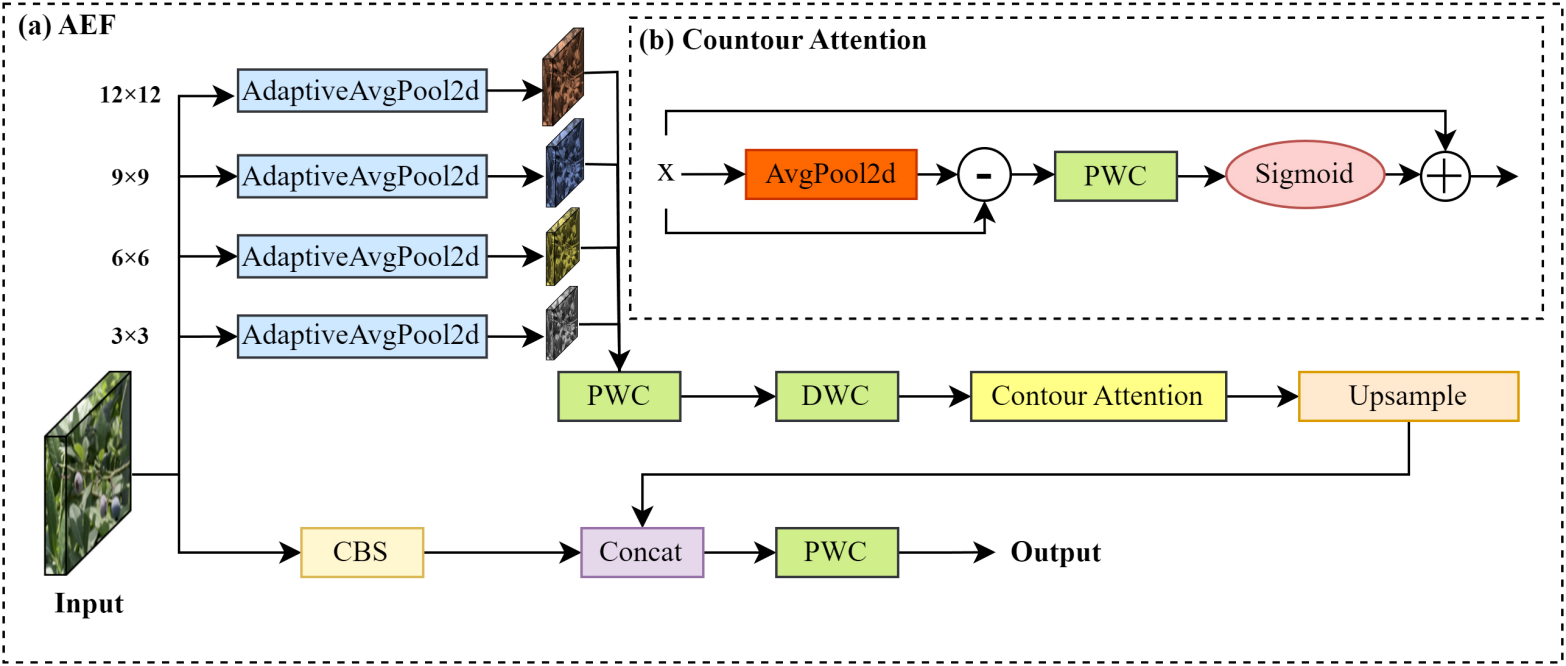
Adaptive edge fusion structure.

As illustrated in **Figure 6a**), the AEF module processes the input image through two parallel pathways. One pathway applies convolution operations to extract initial feature maps, while the other pathway performs multi-scale feature extraction by utilizing adaptive average pooling at various scales to generate feature maps of different resolutions. These multi-scale feature maps enable the model to perceive the fruit edges at multiple levels of detail, particularly providing richer edge features when parts of the fruit are occluded, thereby compensating for the information loss caused by occlusion.

For each scale of the feature maps, the module further employs pointwise convolution (PWC) and Depthwise convolution (DWC) for feature compression before passing them into the contour attention (CA) module, as shown in **Figure 6b**). The CA module first performs average pooling on the feature maps to extract their low-frequency components. It then calculates the difference between the feature maps and their low-frequency components to obtain high-frequency edge information. Specifically, the input feature map *X ∈* $\;R^{C\times H\times W}$, represents a tensor with *C* channels, *H* height and *W* width. By applying a 3×3 average pooling to each channel of the feature map, the low-frequency component *E* is computed. Subsequently, the difference between the input feature *X* and *E* yields the high-frequency component *R*, as shown in Equations 3, 4. This difference effectively highlights the high-frequency components in the image, such as edges and texture details.

(3) $$\begin{array}{c} E_{c,i,j}=\frac{1}{9}\sum_{m=-1}^{1}\sum_{n=-1}^{1}X_{c,i+m,j+n} \end{array}$$

(4) $$\begin{array}{c} R_{c,i,j}=X_{c,i,j}-E_{c,i,j} \end{array}$$

where *c* denotes the channel index, while *i* and *j* represent the spatial position indices of the feature map. The variables *m* and *n* indicate the offsets relative to the current position. Subsequently, a PWC is applied to the edge information *R*, and the weights are adjusted using a sigmoid function to obtain the modified edge information *R′*, as illustrated in Equation 5.

(5) $$\begin{array}{c} R_{c,i,j}^{'}=\sigma (\sum_{d=1}^{C}\sum_{u=-k}^{k}\sum_{v=-k}^{k}W_{c,d,u,v}\cdot R_{d,i+u,j+v}) \end{array}\;$$

where $W_{c,d,u,v}$ represents the weight parameters of the convolution kernel, and *k* denotes the radius of the convolution kernel. The symbol *σ* signifies a linear activation function. The adjusted edge information *R′* is added to the original feature map *X*, resulting in the final enhanced feature map *Y*, as shown in Equation 6.

(6) $$\begin{array}{c} Y_{c,i,j}=X_{c,i,j}+R_{c,i,j}^{'} \end{array}\;$$

This operation enhances edge information, enabling the CA module to extract fruit contours and improve edge perception in complex scenes, overcoming occlusion interference. After processing, all enhanced edge feature maps at different scales are upsampled back to their original sizes and fused through channel concatenation (Concat) operations, integrating multi-scale edge information into a unified feature space. Simultaneously, the features extracted from the other convolutional pathway are also concatenated with the fusion results, combining global and local features to further enhance the model's ability to perceive fruit edge information in complex scenarios. Finally, a PWC is applied to compress and integrate the concatenated feature maps, generating the final output feature map for subsequent blueberry fruit maturity detection tasks.

The proposed AEF can be expressed by Equation 7:

(7) $$\begin{array}{c} \;F_{output}=PWConv(Concat(\{CA(Pool_{i}(F_{conv}))丨i\in S\;\})) \end{array}\;$$

where $F_{conv}$ is to extract the initial features; $Pool_{i}$ represents the adaptive average pooling operation at the *i* -th scale, and *S* represents the target size of each pooling operation (e.g., 3×3, 6×6, 9×9, 12×12, etc.); *Concat* is the channel concatenation of multi-scale features. *PWConv* stands for the 1×1 PWC used to compress the fusion.

The AEF module is designed to mitigate the negative impact of partial occlusion on fruit edge feature extraction. Through its combination of multi-scale feature extraction and a contour attention mechanism, the module enhances visible edge information at different scales. This process helps the model infer the presence of a fruit from incomplete contours, thereby compensating for missing edge details when parts of the fruit are occluded. Furthermore, the module's lightweight design ensures computational efficiency, making it suitable for real-time detection on embedded devices. Therefore, the module improves the model's robustness against partial occlusion and complex backgrounds while maintaining high accuracy, enabling more precise detection of blueberry fruits.

##### 2.3.2.3 Multi-scale neck structure

Due to the small size of blueberry fruit and its vulnerability to distant blur, it is prone to missed detection during the detection process. To address this issue, a MNS is devised to enhance the model's detection capability for objects of various scales, particularly for small ones.

The core of MNS lies in the utilization of feature layers of different scales for information transfer and fusion, generating feature maps enriched with multi-scale features. By adopting a feature pyramid and multi-layer fusion strategy, the MNS extracts and integrates features of different scales, enabling the detection of objects of various sizes, especially small targets, in complex scenarios (Lin et al., 2017).

**Figure 7:**
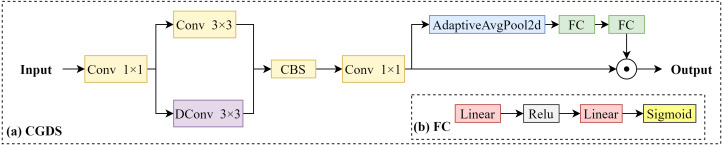
Context guided down-sampling module.

Specifically, this structure acquires multi-level feature maps from the Backbone network, which contain information of varying resolutions and receptive fields. These feature maps allow the model to focus on objects of different sizes while preserving critical spatial details. As shown in the Neck part of **Figure 3**, after passing through the SPPF module, the feature maps are processed step by step, and multi-scale context information is extracted *via* the Context guided down-sampling (CGDS) module (Wu et al., 2021), as illustrated in **Figure 7a**).

The CGDS module plays a crucial role in the feature extraction process. Input features are first compressed using a 1×1 convolution to reduce dimensionality. Next, spatial and contextual information are extracted using standard 3×3 convolution and dynamic convolution (DConv), respectively. The multi-scale features are then fused by the CBS module, followed by further processing with a 1×1 convolution and adaptive average pooling to generate global context information. Finally, a fully connected layer (as shown in **Figure 7b**) generates weight distributions to adaptively adjust the features.

This lightweight design allows the CGDS module to effectively combine local information with global context, ensuring that spatial details are preserved throughout the feature extraction process. As a result, the processed feature maps at each scale are rich in context information, significantly enhancing the network's feature expression and improving its ability to detect small targets and objects of different scales.

##### 2.3.2.4 Shared differential convolution head

In the original YOLOv8 network, each layer feature map is independently convolved to ensure the complete transfer of features. Nevertheless, this design brings about a considerable increase in computation and memory overhead, particularly when multi-scale feature fusion is carried out. To reduce the computational cost while maintaining high detection accuracy, this paper presents a SDCH. By sharing the convolution operation among different feature layers, the proposed method not only effectively reduces the computational redundancy but also guarantees the effectiveness of feature extraction.

**Figure 8:**
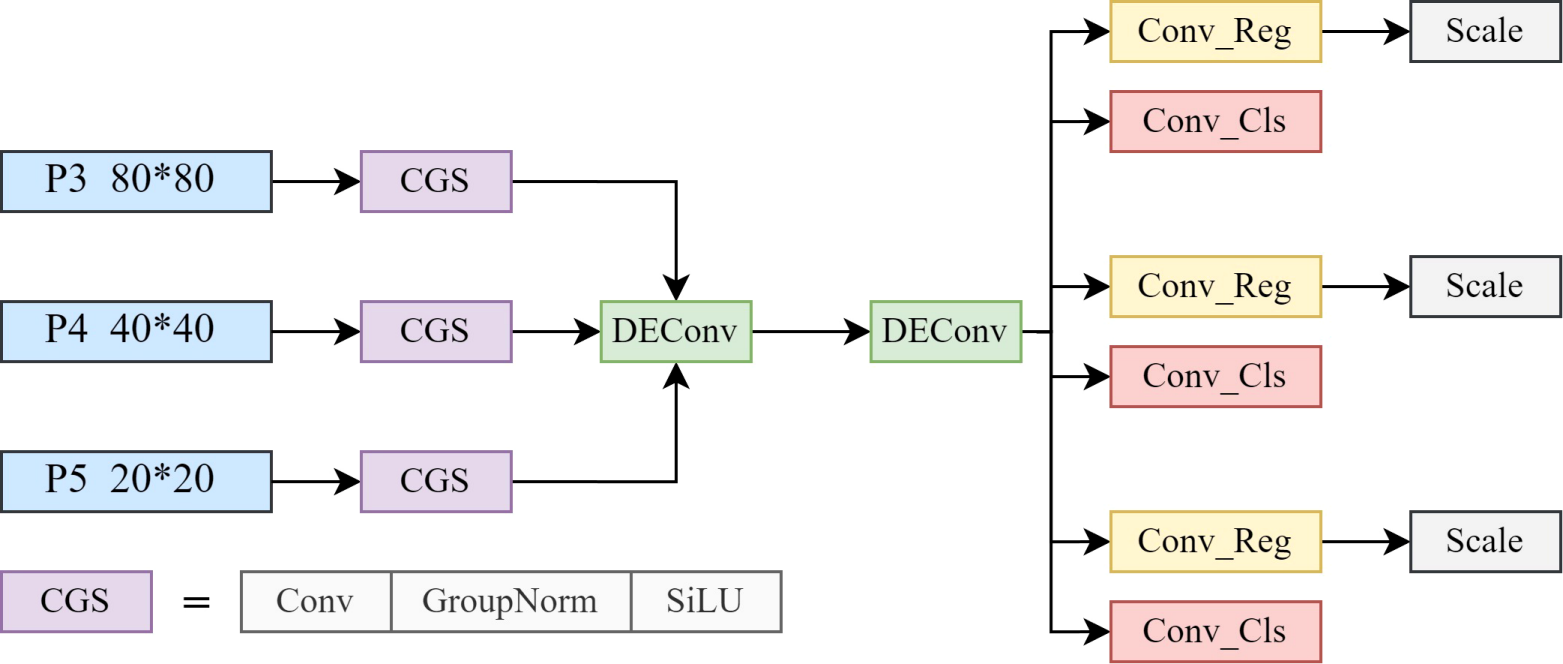
Shared differential convolution head.

As depicted in **Figure 8**, the CGS module, consisting of a convolutional layer (Conv), Group Normalization (GroupNorm), and SiLU activation, is utilized to process input feature layers (P3, P4, and P5).

**Figure 9:**
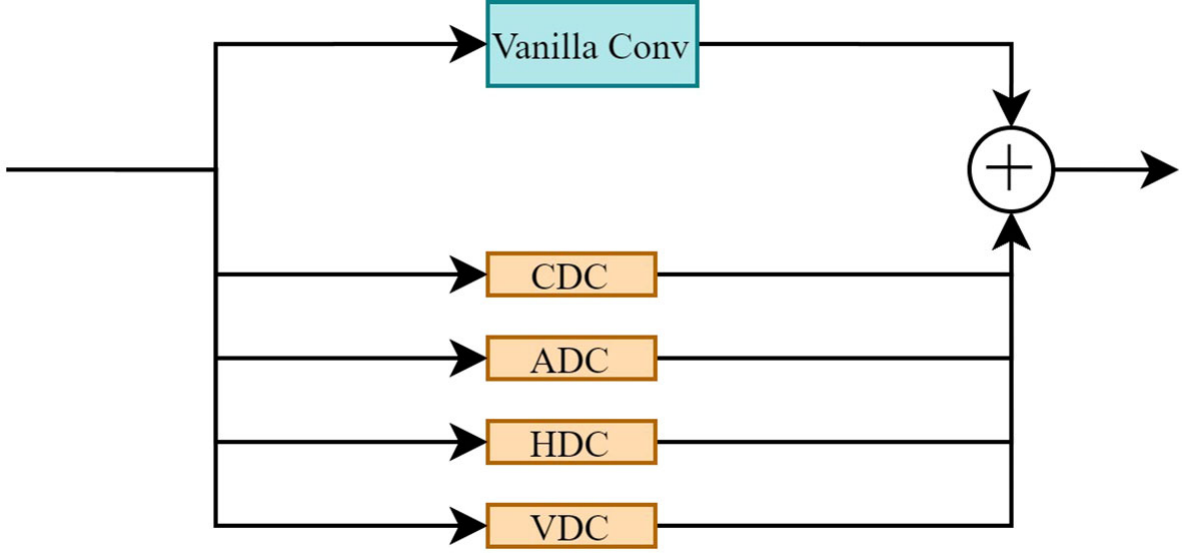
Detail-enhanced convolution structure.

This module adapts the input data to varying feature scales, ensuring consistent feature representation across different levels. The convolutional layer extracts the essential features, while GroupNorm stabilizes the feature distribution, improving robustness and consistency during feature fusion. Finally, the SiLU activation function introduces nonlinearity, enhancing the expressiveness of the extracted features. As has been verified by Tian et al. (2022), the CGS module effectively normalizes and fuses features, significantly improving the model’s detection accuracy, particularly for small objects. The function description is provided in Equation 8.

(8) $$\begin{array}{c} GroupNorm(x)=\frac{x-\mu_{g}}{\sqrt{\sigma_{g}^{2}+\in }}\cdot \gamma +\beta \end{array}$$

where *x* is the input feature map; $\mu_{g}$ and $\sigma_{g}^{2}$ are the mean and variance of *x* over each group, respectively. The *∈* is a small constant to prevent the denominator from becoming zero. γ and β are learnable scaling and offset parameters. The processed feature maps are fed into the shared two-layer detail-enhanced convolution (DEConv), as shown in **Figure 9**, to further fuse the multi-scale features.

DEConv, which integrates a Vanilla Convolution (standard convolution) and a differential convolution structure, effectively captures high-frequency detailed features, such as edges and contours (Chen et al., 2024). Its core encompasses central difference convolution (CDC), angle difference convolution (ADC), horizontal difference convolution (HDC), and vertical difference convolution (VDC), which extract edge information from different directions. By sharing the DEConv, the feature extraction of P3, P4, and P5 is unified, significantly reducing the number of convolutional layers and model parameters while maintaining strong feature representation capabilities.

After DEConv processing, the feature map $F_{all}$ is split into two paths: one path is utilized for the regression prediction $P_{reg}$ of the object position, and the other path is used for the classification prediction $\;P_{cls}$ of the object category. The specific formula is shown in Equation 9:

(9) $$\begin{array}{c} P_{cls}=Conv_{cls}(F_{all})\;\;,P_{reg}=Conv_{reg}(F_{all}) \end{array}$$

After that, the scale operation adjusts the scale of the output features to adapt to the output requirements of different levels, and finally generates the object detection results.

##### 2.3.2.5 Evaluation standard

To evaluate the performance of the proposed model, we utilized commonly used metrics in object detection, including Precision, Recall, Average Precision (AP), and mean Average Precision (mAP). The calculations for these metrics are as shown in Equations 10–13:

(10) $$\begin{array}{c} Precision=TP/\;(\;TP+FP\;)\times 100\% \end{array}$$

(11) $$\begin{array}{c} Recall=TP/(\;TP+FN\;)\times 100\% \end{array}$$

(12) $$\begin{array}{c} AP=\int_{0}^{1}P(Recall)dR \end{array}$$

(13) $$\begin{array}{c} mAP=\frac{1}{n}\;\sum_{i=1}^{n}AP(i)\times 100\% \end{array}$$

where *TP* denotes the true positive defections. *FP* represents false positives and *FN* refers to false negatives. Precision and Recall are used to derive the precision-recall curve, which evaluates the trade-off between these metrics across various thresholds.

Param refer to the total number of learnable parameters in the model, while FLOPs measure the number of floating-point operations required to process a single input image. Both metrics are crucial for evaluating the model's efficiency and computational complexity. Lower values of Parameters and FLOPs indicate a lighter and more efficient model.

Additionally, Counting Accuracy ( ${\text{P}}_{\text{c}}$) and Mean Counting Accuracy ( ${\text{mP}}_{\text{c}}$) were employed to assess the counting results. These metrics are defined as shown in Equations 14, 15:

(14) $$\begin{array}{c} P_{c}=(\;1-|N_{a}-N_{t}|/\;\;N_{t}\;)\times 100\% \end{array}$$

(15) $$\begin{array}{c} mP_{c}=\frac{1}{3}(P_{c_{\;blue}}+P_{c_{\;green}}+P_{c_{\;purple}}) \end{array}$$

In these equations, $N_{a}$ represents the automatically counted value, and $N_{t}$ denotes the true count value. *m* is the number of instances evaluated. Higher values of $P_{c}$ and $mP_{c}$ indicate more accurate counting results.

### 2.4 ByteTrack algorithm

Furthermore, in order to address the challenges associated with manual counting, this study explores an automated counting method based on raw blueberry videos for blueberry fruit counting. The automated counting process was achieved by integrating STF-YOLO with the widely-used Multiple Object Tracking (MOT) method. The actual number of blueberries at each growth stage was obtained through manual counting during video collection.

**Figure 10:**
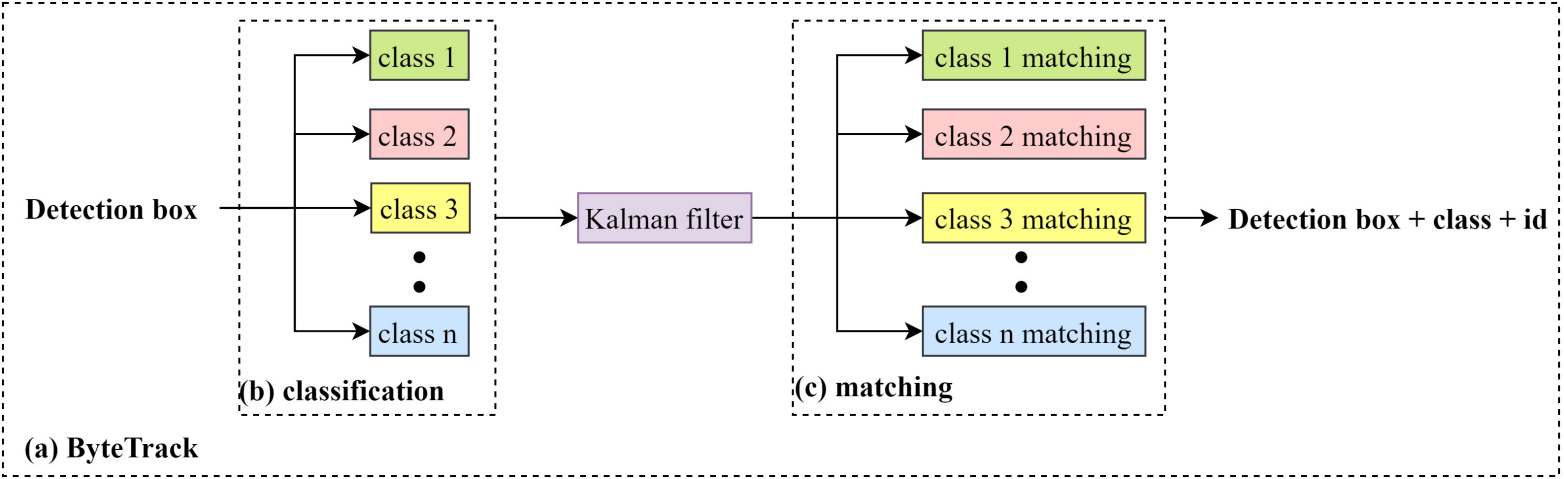
The tracking process of ByteTrack algorithm.

The ByteTrack algorithm enhances the accuracy of object-counting by introducing a data-association technique called Better Tracking-by-Detection (Byte) as illustrated in **Figure 10** (Zhang et al., 2022). Building on STF-YOLO’s precise detections, ByteTrack efficiently links detected blueberry instances across frames, making it particularly effective in high-density scenarios with overlapping fruits and frequent occlusions. By integrating ByteTrack with STF-YOLO, our system combines state-of-the-art detection and robust temporal tracking to deliver a reliable, end-to-end automated blueberry counting solution.

### 2.5 Evaluation standard

To evaluate the performance of the proposed model, we utilized commonly used metrics in object detection, including Precision, Recall, Average Precision (AP), and mean Average Precision (mAP). The calculations for these metrics are as shown in Equations 16–19:

(16) $$\begin{array}{c} Precision=TP/\;(\;TP+FP\;)\times 100\% \end{array}$$

(17) $$\begin{array}{c} Recall=TP/(\;TP+FN\;)\times 100\% \end{array}$$

(18) $$\begin{array}{c} AP=\int_{0}^{1}P(Recall)dR \end{array}$$

(19) $$\begin{array}{c} mAP=\frac{1}{n}\sum_{i=1}^{n}AP(i)\times 100\% \end{array}$$

where *TP* denotes the true positive defections. *FP* represents false positives and *FN* refers to false negatives. Precision and Recall are used to derive the precision-recall curve, which evaluates the trade-off between these metrics across various thresholds.

Param refer to the total number of learnable parameters in the model, while FLOPs measure the number of floating-point operations required to process a single input image. Both metrics are crucial for evaluating the model's efficiency and computational complexity. Lower values of Parameters and FLOPs indicate a lighter and more efficient model.

Additionally, Counting Accuracy ( ${\text{P}}_{\text{c}}$) and Mean Counting Accuracy ( ${\text{mP}}_{\text{c}}$) were employed to assess the counting results. These metrics are defined as shown in Equations 20, 21:

(20) $$\begin{array}{c} P_{c}=(\;1-|N_{a}-N_{t}|\;/\;\;N_{t}\;)\;\times 100\% \end{array}$$

(21) $$\begin{array}{c} mP_{c}\;=\frac{1}{3}\;(\;P_{c_{\;blue}}+P_{c_{\;green}}+P_{c_{\;purple}}\;)\;\#(16) \end{array}$$

In these equations, $N_{a}$ represents the automatically counted value, and $N_{t}$ denotes the true count value. *m* is the number of instances evaluated. Higher values of $P_{c}$ and $mP_{c}$ indicate more accurate counting results.

## 3 Results

### 3.1 Experimental platform and parameter settings

This experiment was conducted using the following hardware and software configurations. The hardware setup included an AMD Ryzen 9 5900X 12-core processor running at 3.70 GHz, paired with an Nvidia GeForce RTX 3090 graphics card and 128 GB of RAM. The operating system used was Windows 11. The software environment comprised PyCharm 2022 as the development environment, Python version 3.12.4, and Torch version 2.3.1. During the experiments, the size of the training images is set to 640×640 pixels, the epoch is 150, the batch size is 8, and the learning rate is 0.01.

### 3.2 Comparative experiment of different models

To validate the detection performance of STF-YOLO more comprehensively, we compared it with nine lightweight or enhanced YOLO variants. As shown in **Table 2**, STF-YOLO demonstrates state-of-the-art performance across multiple key metrics. Under the standard mAP50 metric, STF-YOLO achieves the highest score of 79.7%. It is 2.9%, 3.3%, 3.5%, 2.0%, 3.7%, and 1.9% higher than YOLOv5 to YOLOv11, respectively, and it outperforms YOLO-MIF, MAF-YOLO, and YOLO-SDFM by 2.5%, 2.4%, and 1.6%. Furthermore, STF-YOLO attains the highest precision (82.3%) and recall (72.1%), representing improvements of 0.6% and 3.5 % over YOLOv8, respectively. Crucially, when evaluated under the stricter mAP50–95 metric, which demands higher localization accuracy, STF-YOLO again achieves the top performance with 52.5%, surpassing all other models. This demonstrates that STF-YOLO not only identifies objects accurately but also provides more precise bounding box localization than other advanced variants. Notably, these comprehensive accuracy improvements are achieved with an efficient model size of 2.67 million parameters and a computational cost of 6.7 GFLOPs. This shows that STF-YOLO successfully combines state-of-the-art detection performance with exceptional lightweight efficiency, making it highly suitable for practical agricultural scenarios.

**Table 2:** Performance comparison of different models.

Model	Precision (%)	Recall (%)	mAP50 (%)	mAP50-95 (%)	Params (M)	FLOPS (G)
YOLOv5n	80.8	70.8	76.8	50.7	2.50	7.1
YOLOv6n	81.0	68.8	76.4	50.5	4.23	11.8
YOLOv8n	81.7	68.6	76.2	51.2	3.01	8.1
YOLOv9t	81.9	71.1	77.7	51.3	**1.97**	7.6
YOLOv10n	80.2	69.1	76.0	50.7	2.27	6.5
YOLOv11n	82.0	70.7	77.8	52.1	2.58	**6.3**
YOLO-MIF	81.6	69.1	77.2	51.6	3.01	8.1
MAF-YOLO	80.9	71.4	77.3	50.9	2.99	8.7
YOLO-SDFM	82.2	72.0	78.1	51.6	3.44	8.5
STF-YOLO	**82.3**	**72.1**	**79.7**	**52.5**	2.67	6.7

The bold values represent the best (optimal) result achieved in each respective column.

**Figure 11:**
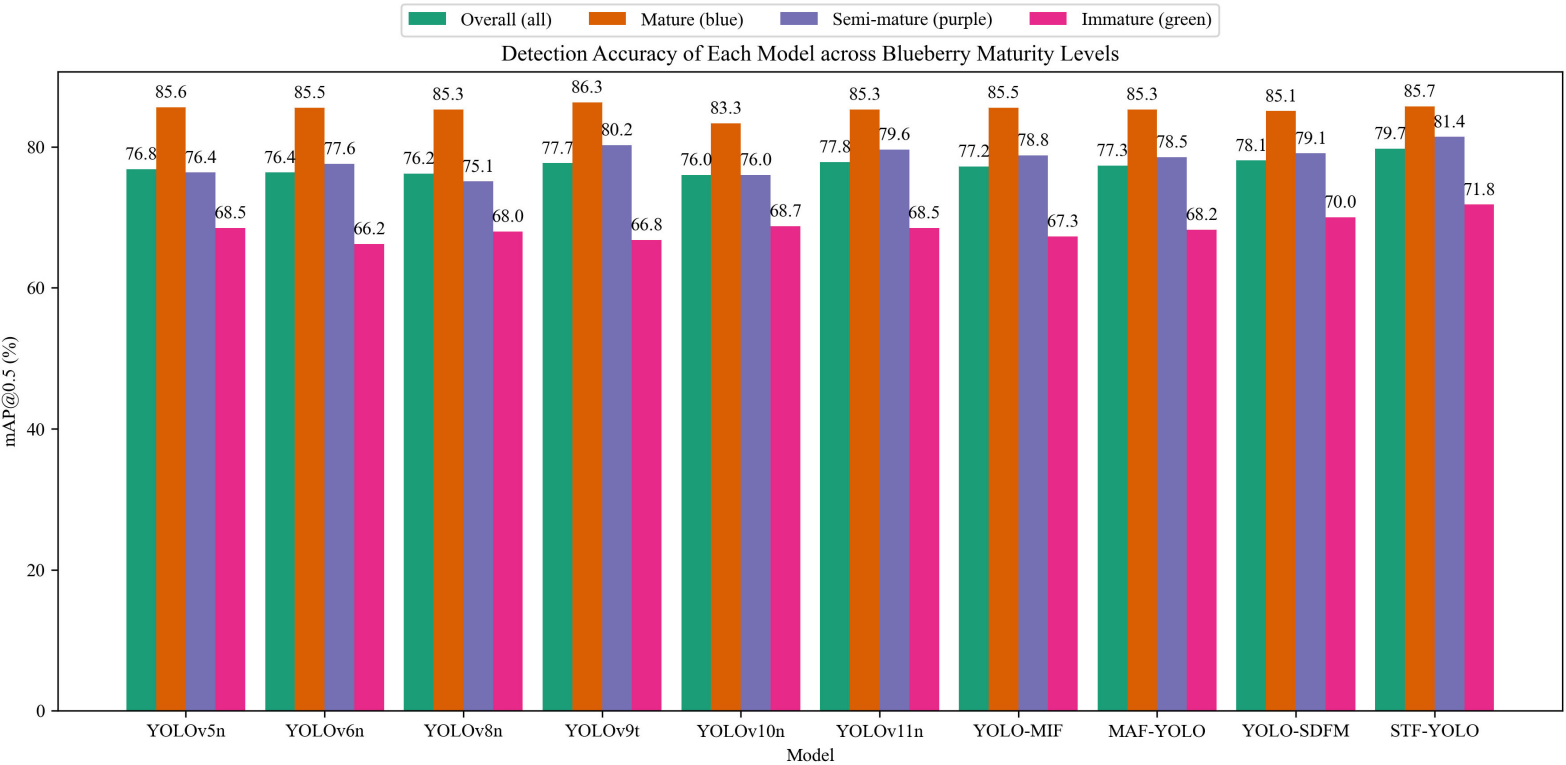
mAP50 for different detection models across blueberry maturity.

**Figure 11** summarizes the mAP50 performance of nine lightweight or enhanced YOLO variants and our proposed STF-YOLO model under four settings—overall (“all”) and three blueberry categories (mature, semi-mature, and immature). STF-YOLO achieves the highest overall mAP50 of 79.7 %, outperforming the next best model, YOLO-SDFM (78.1 %), by 1.6 %. In the mature category, STF-YOLO attains 85.7 % mAP50, just behind YOLOv9t’s 86.3 % yet ahead of the other eight models (83.3 %–85.6 %), demonstrating strong performance when color contrast is high. For semi- mature category, where color cues are subtler, STF-YOLO’s 81.4 % mAP50 exceeds the second-best (YOLOv9t) by 1.2 %, highlighting its sensitivity to intermediate hues. In the immature category—characterized by low contrast against foliage—With a mAP50 of 71.8%, STF-YOLO outperforms YOLO-SDFM by 1.8 %, highlighting its resilience to occlusion and edge ambiguity. These consistent gains across categories confirm that the DSAA, AEF, and MNS modules effectively extract fine-grained, multi-scale features for challenging small-object detection.

### 3.3 Ablation results and insights

To systematically validate the effectiveness of our proposed modules, we conducted a series of ablation experiments, with the results detailed in **Table 3**.

**Table 3:** Ablation experiment outcomes.

YOLOv8n	DSAA	AEF	MNS	SDCH	Precision (%)	Recall (%)	mAP50 (%)	mAP50-95 (%)	FLOPs
✓	×	×	×	×	81.7	68.6	76.2	51.2	8.1G
✓	✓	×	×	×	81.4	70.9	77.2	51.9	7.6G
✓	×	✓	×	×	82.7	69.9	77.9	52.2	7.6G
✓	×	×	✓	×	81.8	69.5	76.8	51.3	9.8G
✓	×	×	×	✓	**84.0**	69.6	77.3	51.8	7.6G
✓	✓	✓	×	×	82.7	71.7	78.2	52.4	7.4G
✓	✓	✓	✓	×	81.7	71.7	78.3	**52.5**	8.3G
✓	✓	✓	✓	✓	82.3	**72.1**	**79.7**	**52.5**	**6.7G**

The bold values represent the best (optimal) result achieved in each respective column.

The baseline YOLOv8n model established a benchmark performance with a mAP50 of 76.2% and a computational cost of 8.1G FLOPs. Initially, we assessed the contribution of each module individually:

The DSAA module replaces the standard C2f structure in YOLOv8 to enhance fine-grained detail perception. Its CATM integrates both spatial and channel self-attention through convolutional query-key interactions, allowing the model to encode contextual dependencies between partially occluded fruit regions. Simultaneously, the CGLU adaptively regulates feature flow by generating gating coefficients that suppress irrelevant background responses. Theoretically, this operation implements a multiplicative feature re-weighting analogous to a selective attention mechanism in human vision, amplifying high-frequency edge cues critical for small-object discrimination. Empirically, the inclusion of DSAA increased mAP50 from 76.2% → 77.2%, confirming that enhanced detail awareness directly improves recognition of maturity-related color–texture transitions.

The AEF module is designed to mitigate information loss caused by leaf occlusion and overlapping fruits. It decomposes each feature map into low-frequency components (global color distribution) and high-frequency components (edge textures) using local average pooling and residual subtraction. The CA mechanism then re-weights the high-frequency responses *via* sigmoid activation, effectively restoring missing contour information. From a signal-processing perspective, AEF acts as a high-pass enhancement operator embedded within the convolutional feature space, selectively amplifying edge gradients associated with blueberry boundaries. This yields stronger gradient flow during backpropagation, improving edge localization. As shown in the ablation results, AEF alone raised mAP50 to 77.9%, and when combined with DSAA, achieved 78.2%, demonstrating complementary improvements in both contour integrity and textural perception.

Blueberries vary greatly in apparent size due to imaging distance and camera angle. To address scale-dependent information loss, the MNS introduces a CGDS mechanism that fuses global contextual features with local receptive fields through dynamic convolution weighting. Theoretically, this structure approximates a hierarchical Laplacian pyramid where each CGDS block adaptively balances spatial detail preservation and contextual abstraction. This enables consistent representation of small and large fruits within a unified feature space. The ablation study shows that while MNS alone increases FLOPs due to multi-scale aggregation, its integration with DSAA + AEF yields more stable recall and mAP50–95 improvements (up to +1.3%) across categories, particularly enhancing recognition of semi-mature fruits whose features exhibit intermediate hues and subtle boundaries.

The SDCH module addresses the redundancy of independent convolutions across feature scales in the YOLOv8 head. By introducing shared convolutional kernels and Group Normalization, it maintains consistent feature representation while significantly reducing parameters (from 3.01 M to 2.67 M). The theoretical foundation lies in parameter sharing and differential feature extraction: the DEConv integrates CDC, ADC, HDC, VDC to capture directional gradients that encode edge orientation and curvature information. This differential representation enhances the model’s sensitivity to geometric variations without expanding network depth. Notably, SDCH not only improved mAP50 by +1.4% compared to the DSAA + AEF + MNS configuration but also reduced FLOPs by 17.6%, validating its effectiveness in achieving a better accuracy–efficiency trade-off.

This hierarchical integration improves both representational power and generalization, allowing STF-YOLO to achieve 79.7% mAP50, outperforming all compared models while remaining lightweight. The improved mAP50-95 (52.5%) demonstrates enhanced localization precision, which theoretically reflects better alignment of predicted bounding boxes with the ground truth due to improved feature discrimination.

In conclusion, the four novel modules collectively transform the baseline YOLOv8 architecture into a fine-grained, scale-adaptive, and computationally efficient detection framework. The improvements are theoretically grounded in enhanced spatial–spectral feature representation and empirically validated through significant gains in precision, recall, mAP50 and mAP50–95 under real-world conditions.

### 3.4 The effect of the model on video counting

**Figure 12:**
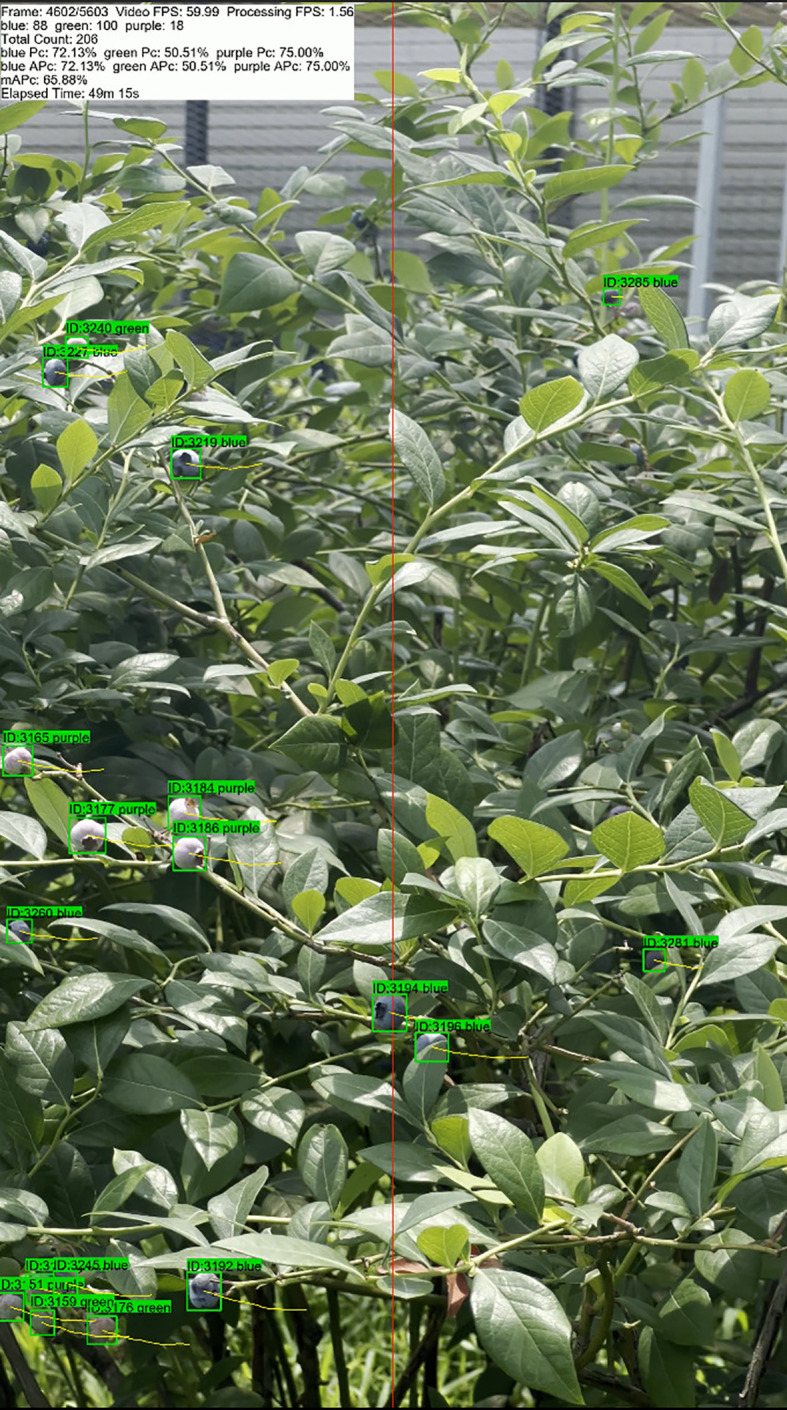
Video counting effect.

**Figure 12** and **Table 4** illustrate the blueberry video counting results and the counting accuracy of STF-YOLO combined with ByteTrack, respectively. In Video 1, the model achieved an $m{\text{P}}_{\text{c}}$ of 69.07%, a ${\text{P}}_{\text{c}}$ of 73.74% for blue category, and a ${\text{P}}_{\text{c}}$ of 72.55% for purple category. These results indicate that the model has a high recognition capability in scenes where the fruit color features are relatively clear. However, the ${\text{P}}_{\text{c}}$ for green category is only 60.92%, demonstrating a decrease in counting accuracy when the color contrast between the fruit and the background is minimal. In Video 2, the model's $m{\text{P}}_{\text{c}}$ increased to 74.94%, with a ${\text{P}}_{\text{c}}$ of 86.07% for blue category and 79.17% for purple category. Despite these improvements, green fruits still exhibit a low ${\text{P}}_{\text{c}}$ of 59.60%, highlighting persistent challenges in counting fruits with less distinct color features. Conversely, in Video 3, the model reached an $m{\text{P}}_{\text{c}}$ of 73.45%, and the ${\text{P}}_{\text{c}}$ for green category improved to 72.22%. This enhancement may be attributed to the relatively brighter background in Video 3 compared to Videos 1 and 2 (as shown in **Supplementary Figure S6**), which increases the color contrast between green category and the background.

**Table 4:** Results of counting blueberries in different videos.

Video	m $P_{c}$(%)	$P_{c_{\;blue}}(\%)$	$P_{c_{\;purple}}(\%)$	$P_{c_{\;green}}(\%)$
1	69.07	73.74	72.55	60.92
2	74.94	86.07	79.17	59.60
3	73.45	76.71	71.43	72.22
all	72.49	78.84	74.38	64.24

**Figure 13:**
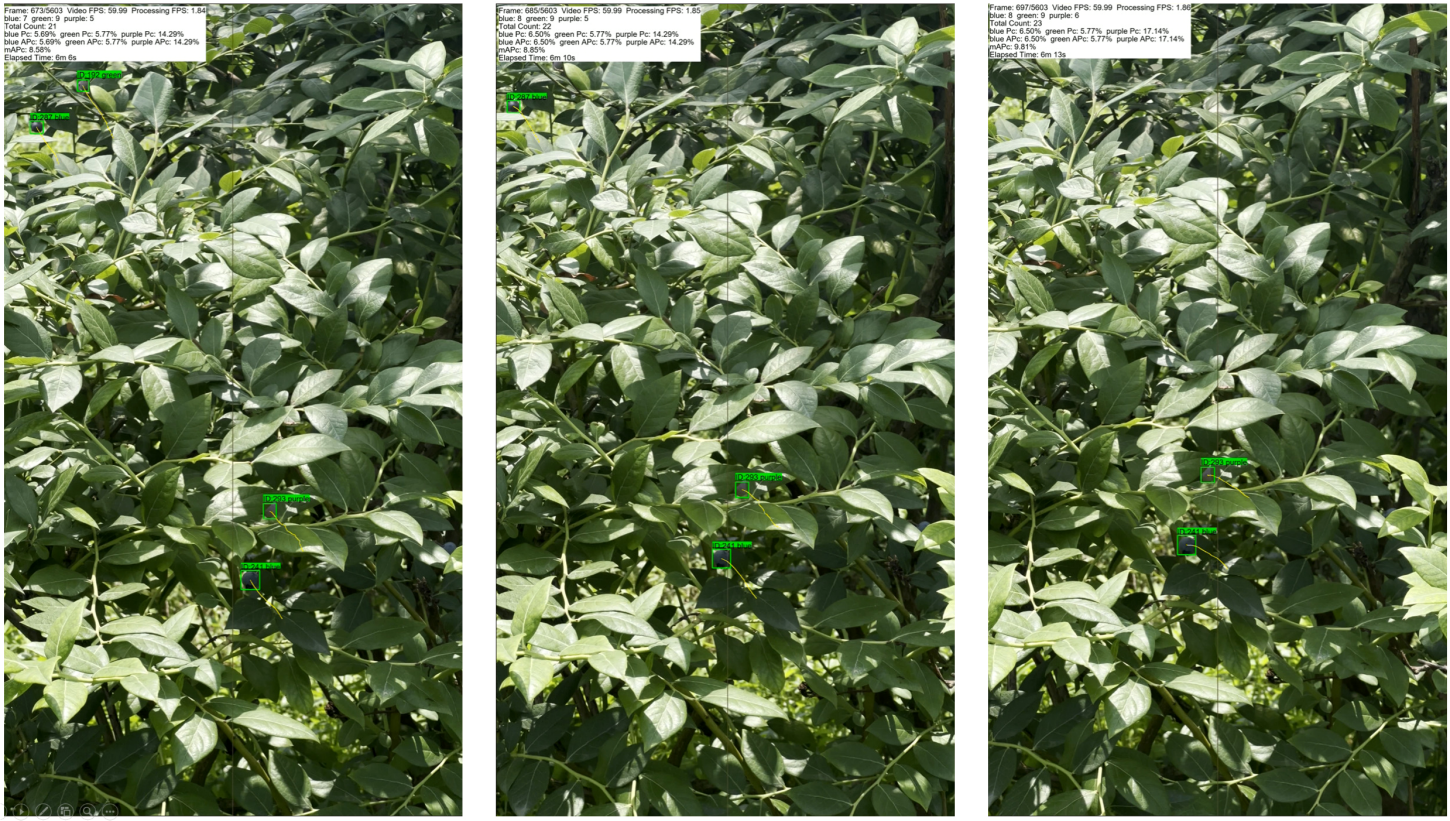
Frame-by-frame display.

As shown in the **Table 4** and **Figure 13**, The counting process of the target tracking algorithm is shown frame by frame, and all the statistics can be clearly seen in the upper left corner. The method integrated STF-YOLO with ByteTrack achieved an m $P_{c}$ of 72.49%. Overall, the proposed method demonstrates strong robustness and effectiveness in blueberry fruit detection and counting across different video scenes. It effectively adapts to varying lighting conditions and background contrasts, maintaining high accuracy and reliable performance. The synergy between STF-YOLO’s precise detection capabilities and ByteTrack’s robust tracking algorithm ensures accurate counting results, even in complex and dynamic environments.

Although traditional manual counting methods may produce more accurate estimates than the results of this study, they usually rely on experienced growers and are only applicable to small-scale orchards. This makes long-term, large-scale, continuous monitoring impractical. By contrast, the automatic counting approach that combines STF-YOLO and the ByteTrack algorithm is objective and repeatable. This significantly reduces labor costs and makes it particularly suitable for deployment in blueberry orchards in real production environments. Furthermore, the model could be improved further — narrowing the gap with manual estimation — by incorporating diverse training data and iteratively refining the algorithm structure. Therefore, the proposed method is highly practical and scalable, effectively addressing the needs of modern agricultural production and precision orchard management.

**Figure 14:**
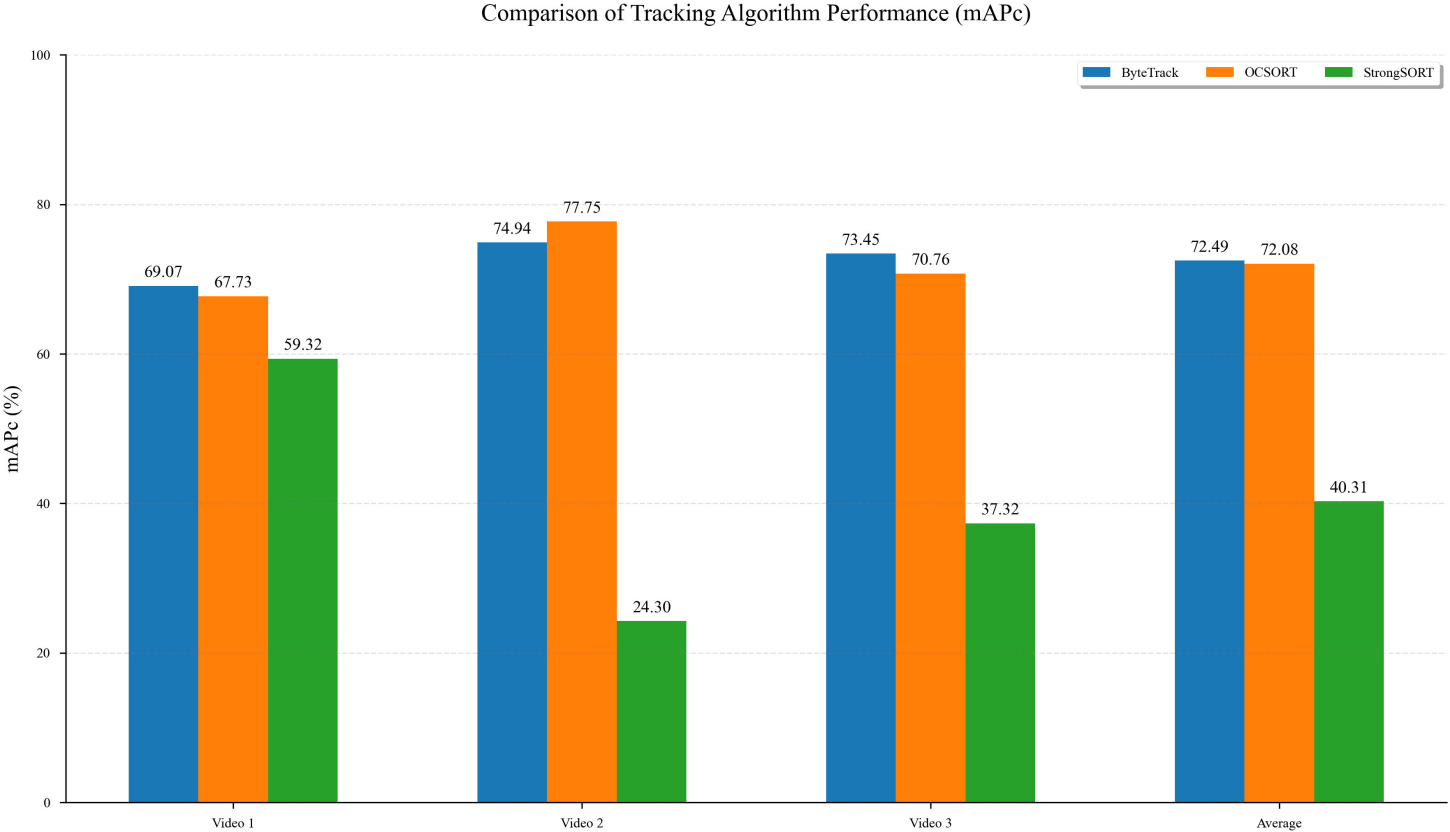
Performance evaluation of different tracking algorithms for object counting.

To evaluate the effectiveness of various multi-object tracking algorithms for the counting task, we selected three mainstream algorithms: ByteTrack, OCSORT, and StrongSORT. These were tested on three video datasets with varying levels of scene complexity. The experimental results are presented in **Figure 14**.

As shown in **Figure 14**, the ByteTrack algorithm demonstrated the best overall performance and stability. It achieved $m{\text{P}}_{\text{c}}$ scores of 69.07%, 74.94%, and 73.45% on the three video test sets, respectively, with an average $m{\text{P}}_{\text{c}}$ of 72.49%, the highest among all algorithms. This indicates that ByteTrack possesses strong robustness in handling challenges such as object occlusion and dense crowds across different scenarios.

The OCSORT algorithm also showed competitive performance, with an average $m{\text{P}}_{\text{c}}$ of 72.08%, which is very close to that of ByteTrack. Notably, OCSORT achieved the highest individual score of 77.75% on Video 2, highlighting its advantages in specific scenarios. However, its performance on the other videos was slightly lower than ByteTrack's, suggesting a minor lack of consistency.

In contrast, the StrongSORT algorithm's performance was unsatisfactory in this experiment. Its average $m{\text{P}}_{\text{c}}$ was only 40.31%, significantly lower than the other two algorithms. A substantial performance degradation was observed, particularly in Video 2 and Video 3, indicating that the algorithm has limited tracking and counting capabilities when dealing with complex, dynamic scenes or frequent object interactions, leading to issues like ID switches or track losses.

In summary, based on its high accuracy and stability, the ByteTrack algorithm is identified as the most suitable tracking algorithm for the target counting task in this evaluation.

### 3.5 Model comparison experiments on different datasets

**Figure 15:**
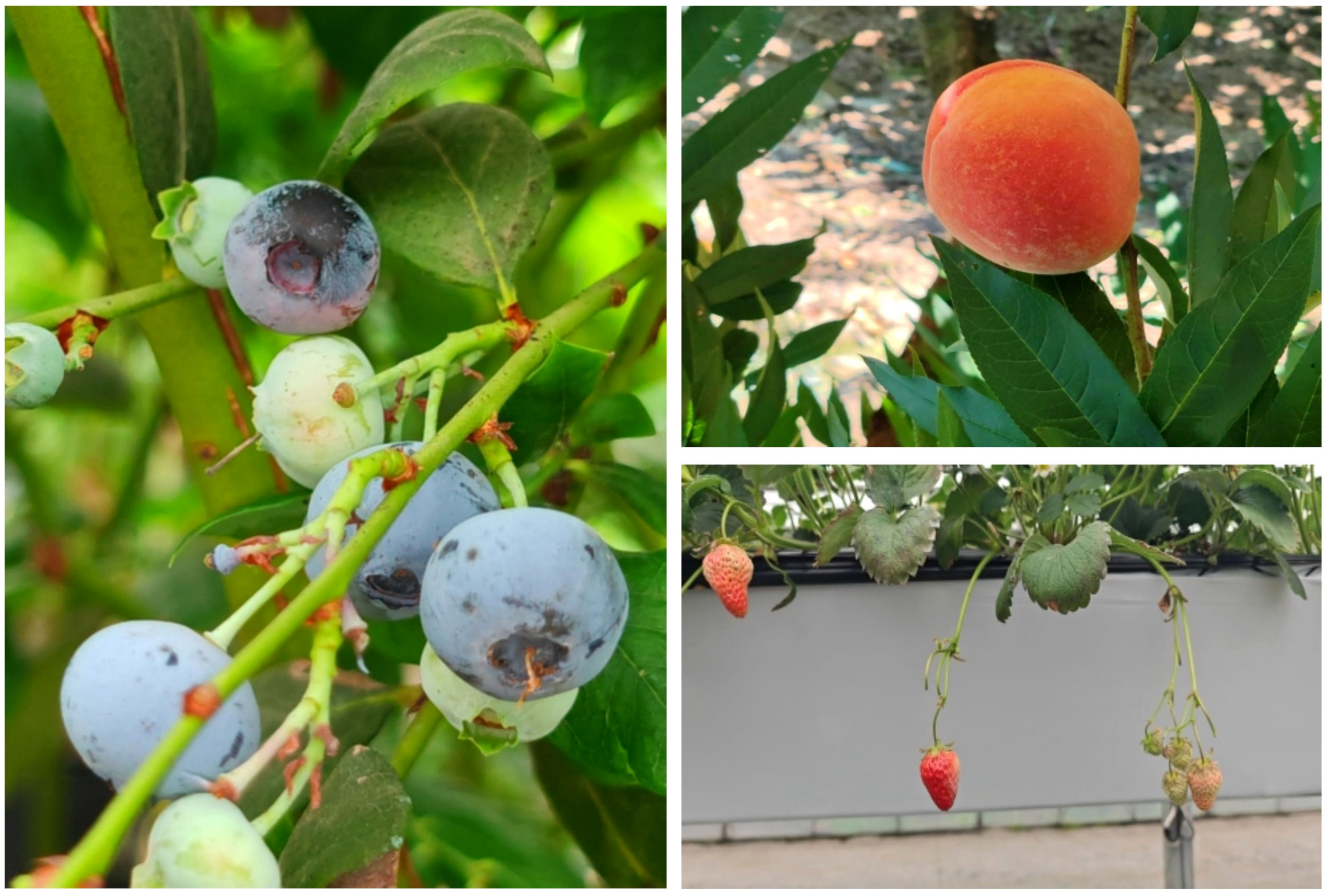
MegaFruit datasets.

In order to further evaluate the generalization capability of the proposed STF-YOLO model, extensive experiments were conducted on two distinct datasets. MegaFruit and PASCAL VOC2007. MegaFruit is a substantial, high-quality dataset of fruit, comprising three subsets: strawberry, blueberry and peach as showed in **Figure 15**. Strawberry comprises 20,242 images. Blueberry comprises 2,540 images, and the peach contains 2,400 images. Conversely, the PASCAL VOC 2007 dataset is a widely used standard in computer vision research. It contains 20 categories of objects, ranging from animals and vehicles to everyday items. This extensive range provides a robust basis for evaluating the generalization and versatility of object detection algorithms.

We have also introduced various models from the YOLO series for comparison purposes. As shown in **Table 5**, the STF-YOLO model achieved the best overall performance on the MegaFruit dataset. Of these, it attained a mAP50 of 91.6% for peaches, 70.5% for strawberries and 90.6% for blueberries. It is worth noting that the blueberry subset was captured at a close range of 20 cm–30 cm with a focus on individual clusters (local views), whereas our own blueberry dataset was recorded from 80 cm–100 cm with a focus on full plants or wider angles. These factors result in higher image resolution and simpler backgrounds in MegaFruit, contributing to its elevated precision. The efficacy of the proposed method is further evidenced by its superior performance compared to other YOLO variants, including YOLOv8n, YOLOv9n, YOLOv10n and YOLOv11n.

**Table 5:** Results of different models on MageFruits dataset.

Model	Mage_peach	Mage_strawberry	Mage_blueberry
YOLOv8n	90.8%	**70.5%**	90.2%
YOLOv9t	91.3%	68.7%	90.4%
YOLOv10n	90.2%	68.3%	89.6%
YOLOv11n	91.5%	70.2%	90.1%
STF-YOLO	**91.6%**	**70.5%**	**90.6%**

The bold values represent the best (optimal) result achieved in each respective column.

**Figure 16:**
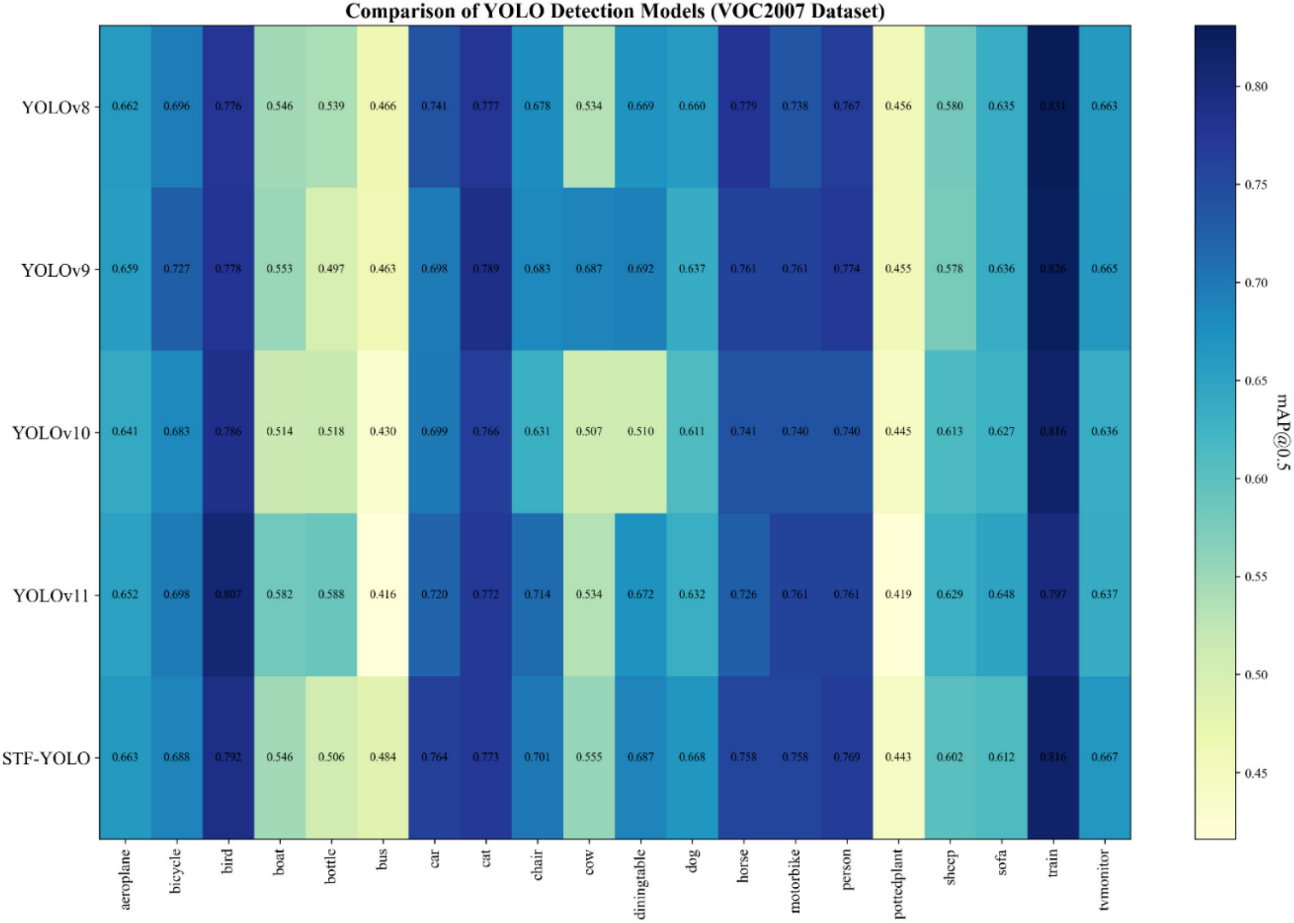
VOC2007 dataset heatmap.

Additionally, when evaluated on the more challenging PASCAL VOC2007 benchmark (see **Table 6**), STF-YOLO still maintained its leading position, reaching a mAP50 of 66.3%, outperforming all the other compared models. The model also achieved a precision of 78.1% and a recall of 50.9%. The class-wise heatmap in **Figure 16** further highlights STF-YOLO's consistently high performance across all 20 VOC categories, especially in the categories of bicycle, bird, dog, horse and motorbike, without the pronounced fluctuations seen in the baseline models. This consistent performance underscores the model's superior generalization capabilities, confirming its applicability to both specialized agricultural detection tasks and broader detection scenarios.

**Table 6:** Results of different models on VOC2007 dataset.

Model	Precision (%)	Recall (%)	mAP50
YOLOv8n	77.3	51.3	66.2
YOLOv9t	75.3	**52.1**	65.9
YOLOv10n	**79.2**	46.7	64.1
YOLOv11n	74.4	51.6	65.2
Ours	78.1	50.9	**66.3**

The bold values represent the best (optimal) result achieved in each respective column.

## 4 Discussion

### 4.1 The necessity of progressing from detection and counting to yield prediction and decision support

In modern blueberry production, the ultimate value of technology lies in its ability to inform management decisions. Traditional yield estimation methods suffer from significant errors due to a lack of objective data support. Therefore, transforming fruit detection and counting technology from purely academic metrics into actionable information that supports decision-making serves as a crucial bridge connecting research and practice. The core contribution of this study lies in developing a lightweight model (STF-YOLO) capable of high-precision detection and counting of blueberries at varying stages of ripeness under complex field conditions, laying the most critical foundation for constructing dynamic yield prediction models.

Placing our research within the existing literature reveals that automated fruit detection and counting represent a research hotspot in the field of yield prediction. Previous studies have achieved significant progress across multiple crops. For instance, Li et al. (2023) successfully combined YOLOv5 and ByteTrack algorithms for video stream counting of dragon fruit flowers, green fruits, and red fruits. Similarly, Du et al. (2024) employed an optimized YOLOv5s and an improved DeepSort algorithm for video tracking and counting of green peppers, effectively addressing challenges such as color similarity and severe occlusion. These studies all achieved favorable results and collectively demonstrate that the “detection + tracking” approach based on the YOLO framework represents the current mainstream and effective technical route.

However, compared to crops like dragon fruit or green peppers with relatively distinct features and larger volumes, blueberries pose greater detection challenges due to their small size, high density, and severe overlapping. Mainstream methods experience significant performance degradation when directly applied to targets like blueberries. The STF-YOLO model proposed in this study significantly enhances feature extraction capabilities for small, edge-blurred objects by integrating modules like DSAA and AEF. It demonstrates outstanding performance among lightweight models of its kind, confirming its superiority in handling such challenging targets.

Furthermore, while many studies (e.g., YOLO-Granada proposed by Zhao et al. (2024) proposed YOLO-Granada) focus on model lightweighting, this study maintains leading detection accuracy while compressing model parameters to 2.67M and reducing computational load to 6.7 GFLOPs—critical for future deployment on edge computing devices. Unlike most studies detecting only ripe fruits, our model distinguishes three maturity stages simultaneously. Its robust generalization capability is validated on the public MegaFruit dataset, demonstrating the universality of our model improvements.

Despite these positive outcomes, several limitations remain. First, the current dataset suffers from limited scale and diversity. Furthermore, as discussed, the dataset exhibits a significant class imbalance (approx. 2:2:1 for mature: immature: semi-mature), with an under-representation of the 'semi-mature' (purple) category. This is because semi-mature fruits have the shortest existence period. Second, as noted in numerous studies (e.g., Du et al., 2024), inferring total fruit counts in 3D space from 2D images presents inherent challenges. Third, severe occlusions remain a primary cause of counting errors. While our method achieves an average counting accuracy of 72.49% when combined with the ByteTrack algorithm, it still falls short of human counting precision. Finally, the accuracy of converting “counts” to “yield” depends on sampling and weighing.

To address these limitations, future work will focus on: constructing larger datasets that are more balanced across different maturity stages and captured throughout the entire growing season to enhance model robustness under varying lighting and weather conditions; exploring the integration of multi-view geometry or 3D reconstruction techniques to fundamentally tackle occlusion issues; and incorporating technologies like multispectral imaging to non-destructively estimate fruit volume and weight, thereby improving the accuracy of final yield predictions.

The ultimate vision for this research framework is its deployment on automated orchard patrol robots or fixed monitoring nodes to collect orchard data periodically (e.g., weekly). The collected time-series count data will be combined with environmental sensor data (temperature and light intensity) to train robust yield prediction models. These models can generate intuitive decision dashboards for farmers, such as: “Area A is projected to reach peak harvest within 7–10 days, with an estimated yield of 100 kilograms.” Such guidance will facilitate human resource allocation, harvesting tool scheduling, and sales planning. It will transform blueberry production from traditional, experience-dependent practices into a new era of data-driven precision smart agriculture.

### 4.2 The influence of different number of subsample blocks on performance

As shown in **Table 7**, we conducted an ablation study on the number of CGDS sub-sampling blocks to evaluate their effect on the overall performance of STF-YOLO. CGDS are the key components of the MNS structure. The various configurations (CGDS = 0-5) are illustrated in **Supplementary Figures S1–S5** and were each tested in a separate experiment. The results indicate that initially, incrementally adding CGDS blocks boosts precision, recall, and mAP; however, beyond a critical point, this yield diminishing returns or even degraded performance. Specifically, the model with CGDS set to 3 achieves the best balance of accuracy (the highest mAP, precision, and recall) and efficiency, with just 2.67 million parameters and 6.7 GFLOPs. Increasing CGDS to 4 or 5 markedly increases computational complexity without improving accuracy. Therefore, CGDS = 3 is identified as the optimal MNS configuration, striking the ideal balance between performance and efficiency.

**Table 7:** Ablation experiment for the number of CGDS.

Models	Precision %	Recall %	map50 %	Params	Flops
STF-YOLO(CGDS = 0)	81.2	70.8	77.7	**2.25M**	**6.1G**
STF-YOLO(CGDS = 1)	81.6	70.8	78.2	2.26M	6.2G
STF-YOLO(CGDS = 2)	82.0	72.0	79.0	2.34M	6.5G
STF-YOLO(CGDS = 3)	**82.3**	**72.1**	**79.7**	2.67M	6.7G
STF-YOLO(CGDS = 4)	81.4	71.2	78.7	2.75M	7.0G
STF-YOLO(CGDS = 5)	81.9	71.7	79.0	3.08M	7.2G

The bold values represent the best (optimal) result achieved in each respective column.

### 4.3 Model detect ability under various complex backgrounds

In the preceding sections, various quantitative analyses and evaluations were conducted on the detection and counting of blueberry maturity. In order to further evaluate the model's robustness in handling complex backgrounds such as low color contrast, occlusions, and overlaps were also examined.

**Figure 17:**
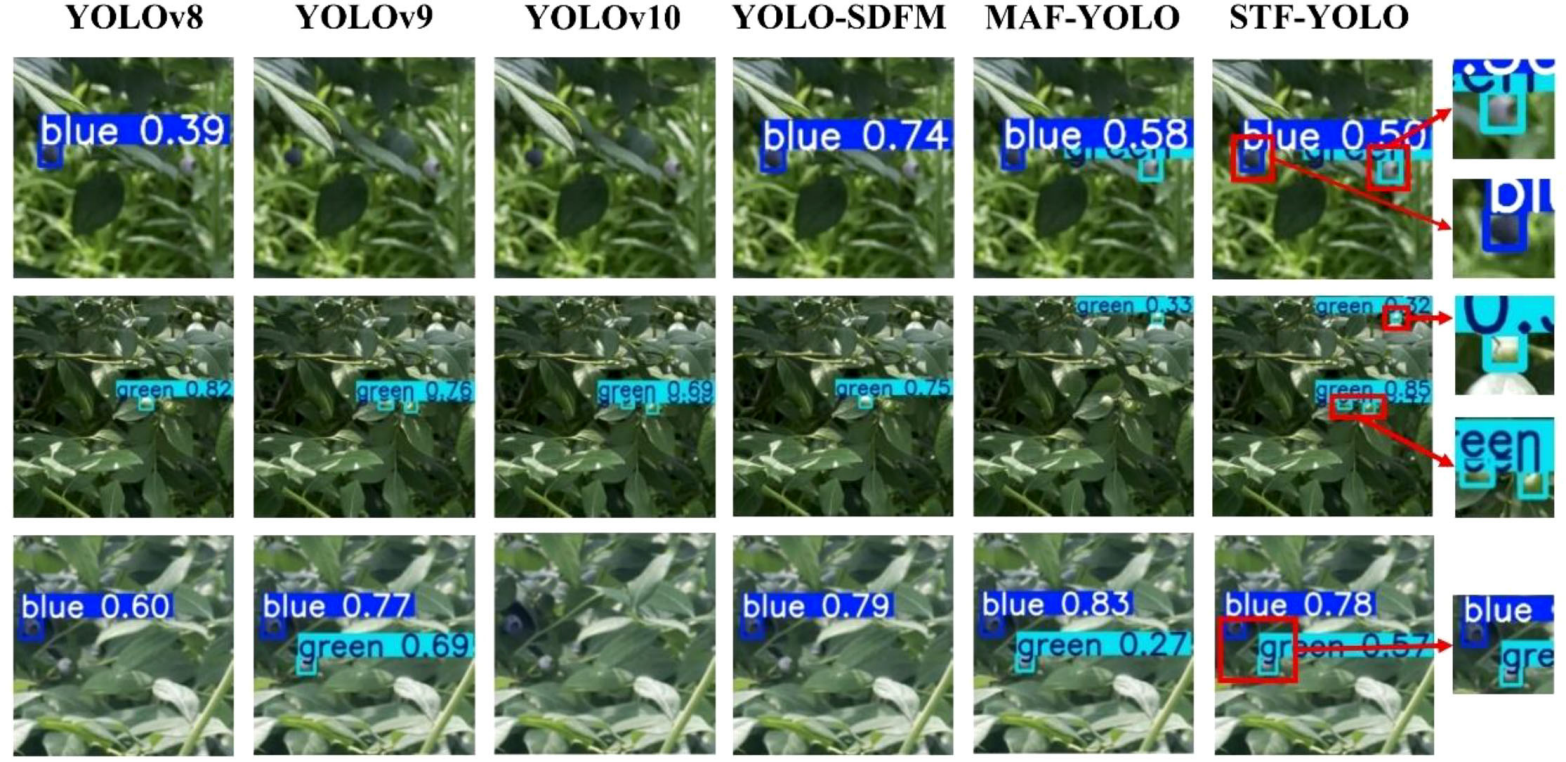
Comparison of detection performance for green immature blueberries and background leaf camouflage scenarios.

As shown in **Figure 17**, the environment is characterized by dense vegetation and immature blueberries. The blueberries' green coloration closely resembles that of the surrounding leaves, which can pose a challenge in blueberry detection and counting. All baseline models, including the improved ones, performed poorly in this scenario, failing to distinguish blueberries from leaves and resulting in missed detections. Only MAF-YOLO successfully handled two of these cases. In contrast, the enhanced STF-YOLO model, leveraging its optimized color perception and discrimination capabilities, significantly improved the detection rate of low-color-difference green blueberries in such scenarios.

**Figure 18:**
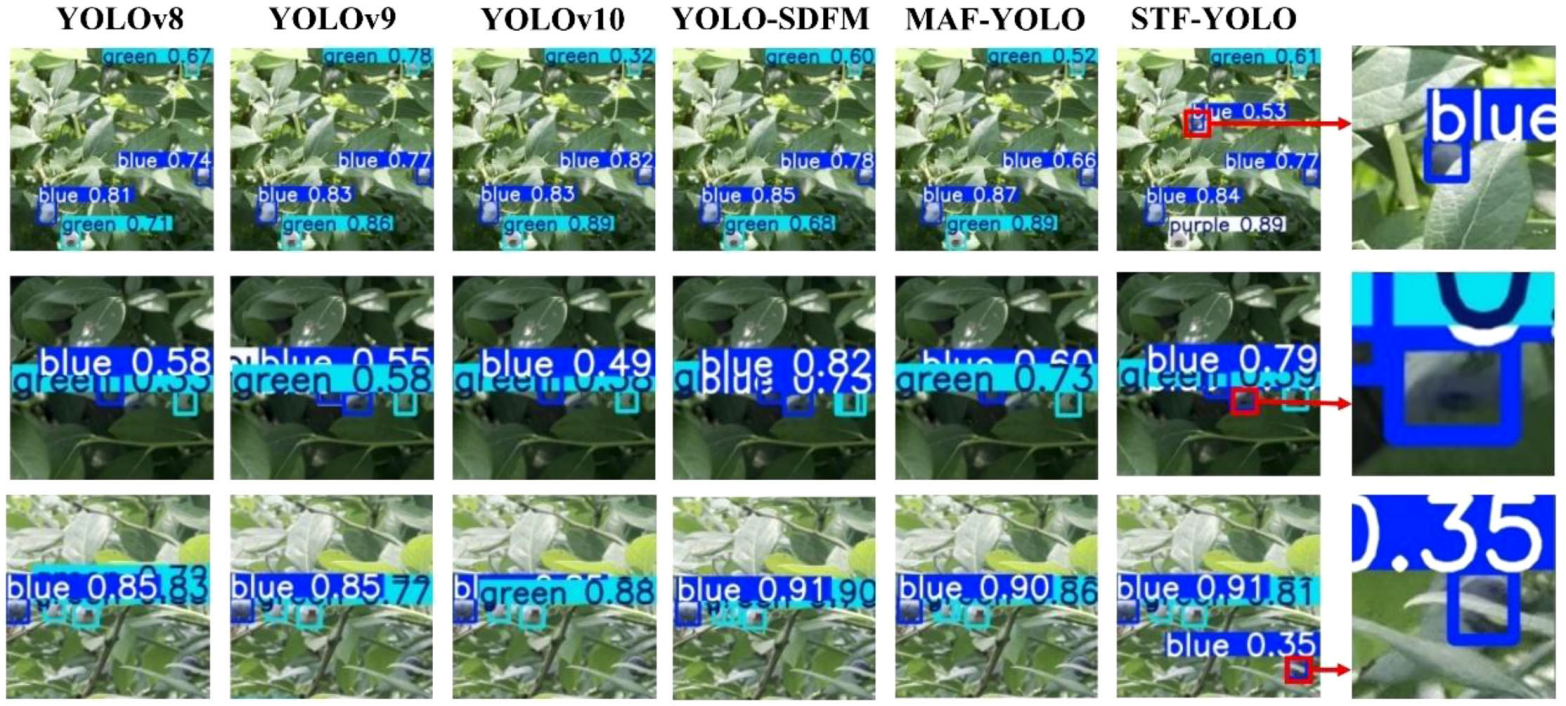
Comparison of detection performance in scenarios with significant fruit occlusion.

**Figure 18** illustrates the detection capabilities of various models in scenario (b), where the environment is characterized by fruits severely obstructed by leaves, branches, or other fruits. This common challenge often leads to missed detections and a substantial drop in bounding-box accuracy for many object detectors. As seen in the figure, baseline models like YOLOv8 and even more advanced versions such as YOLOv9 and YOLOv10, struggle significantly with these occluded targets (e.g., observe the bottom row, where blue blueberries are heavily obscured). Conversely, our proposed STF-YOLO model consistently demonstrates superior performance. Thanks to its enhanced feature extraction and contextual understanding capabilities, STF-YOLO effectively detects partially occluded fruits, showcasing greater reliability and robustness in such complex scenarios compared to other state-of-the-art methods.

**Figure 19:**
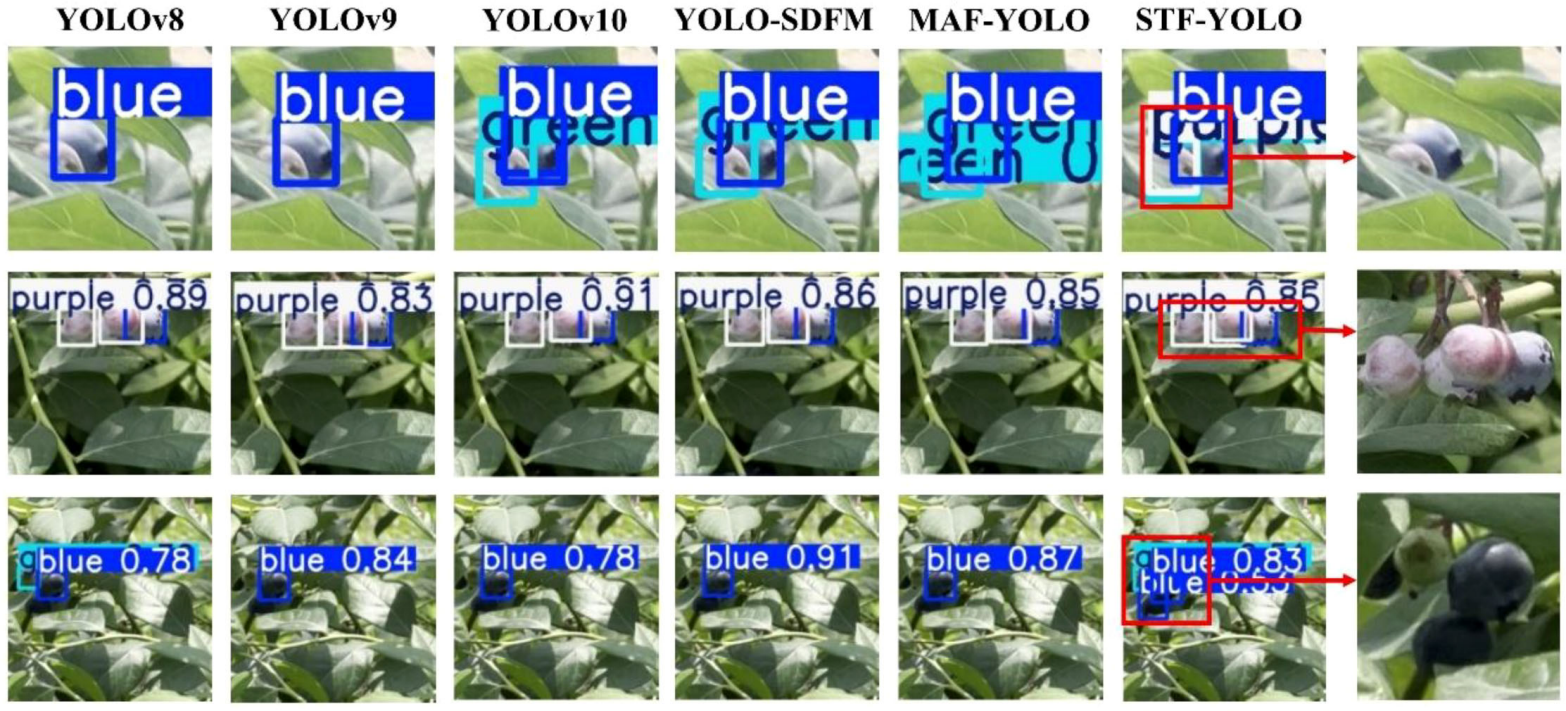
Comparison of detection performance in scenarios with dense and overlapping blueberry clusters.

**Figure 19** presents the challenge of accurately detecting multiple blueberry fruits that are in close proximity or are partially overlapping. As visualized in the comparison, this dense clustering causes most baseline models to fail. These models often mistakenly merge multiple distinct fruits into a single, inaccurate bounding box (e.g., as seen in the top and middle rows) or fail to detect all instances within the cluster. In sharp contrast, our proposed STF-YOLO model demonstrates superior separation capabilities. As highlighted in the enlarged sections, it successfully distinguishes and applies individual bounding boxes to each overlapping blueberry, thereby maintaining high detection accuracy and performance in densely packed conditions.

### 4.4 Direction for improvement

Although the proposed STF-YOLO model demonstrates outstanding blueberry detection performance across various complex scenarios, there remains room for further optimization.

**Figure 20:**
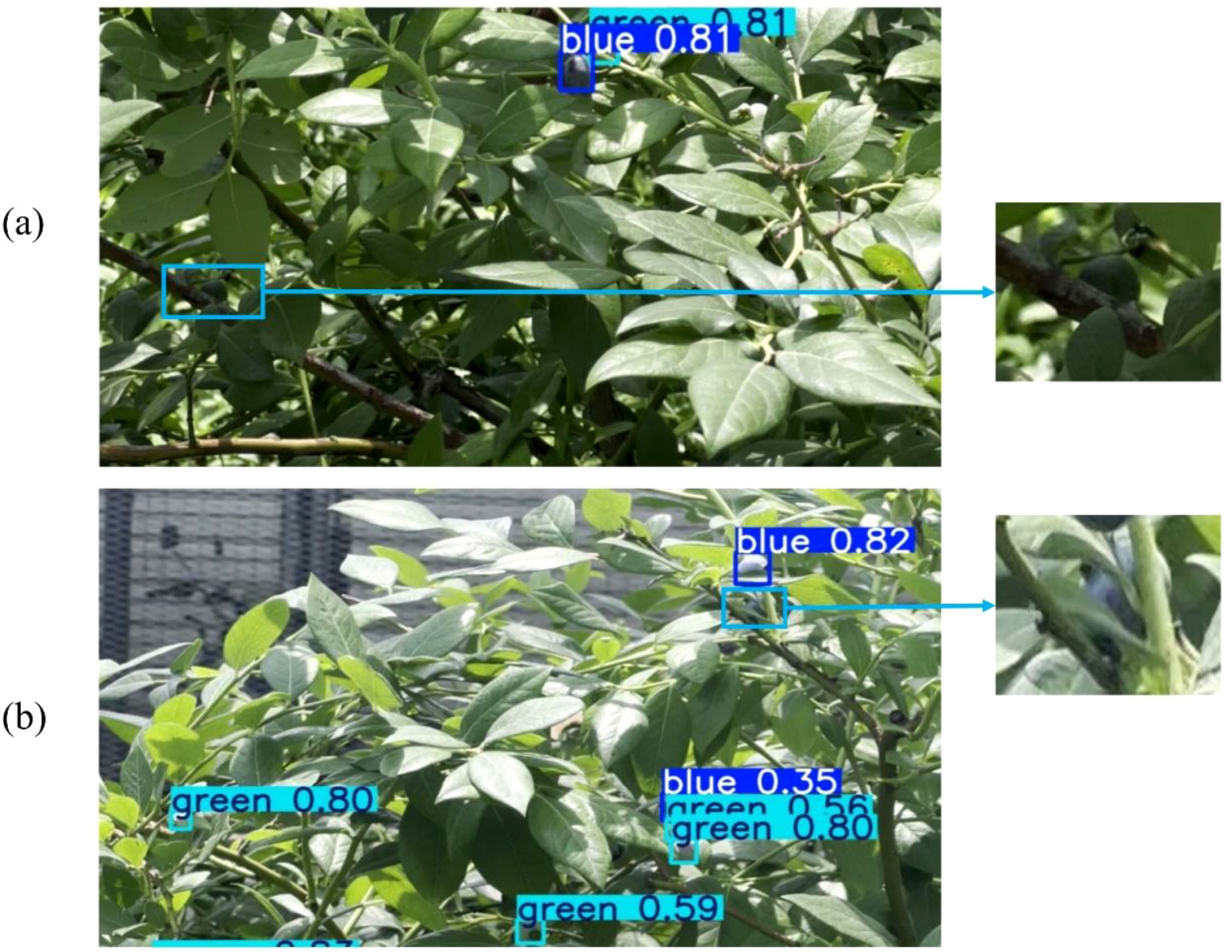
Failure detection phenomenon in extremely complex background: **(a)** Missed detection due to dim lighting; **(b)** Missed detection caused by blurred boundary and severe occlusion.

First, false negatives persist under extremely challenging conditions. For instance, in extremely low-light environments, the model may fail to detect objects due to its inability to extract effective features (as shown in **Figure 20a**).

**Figure 21:**
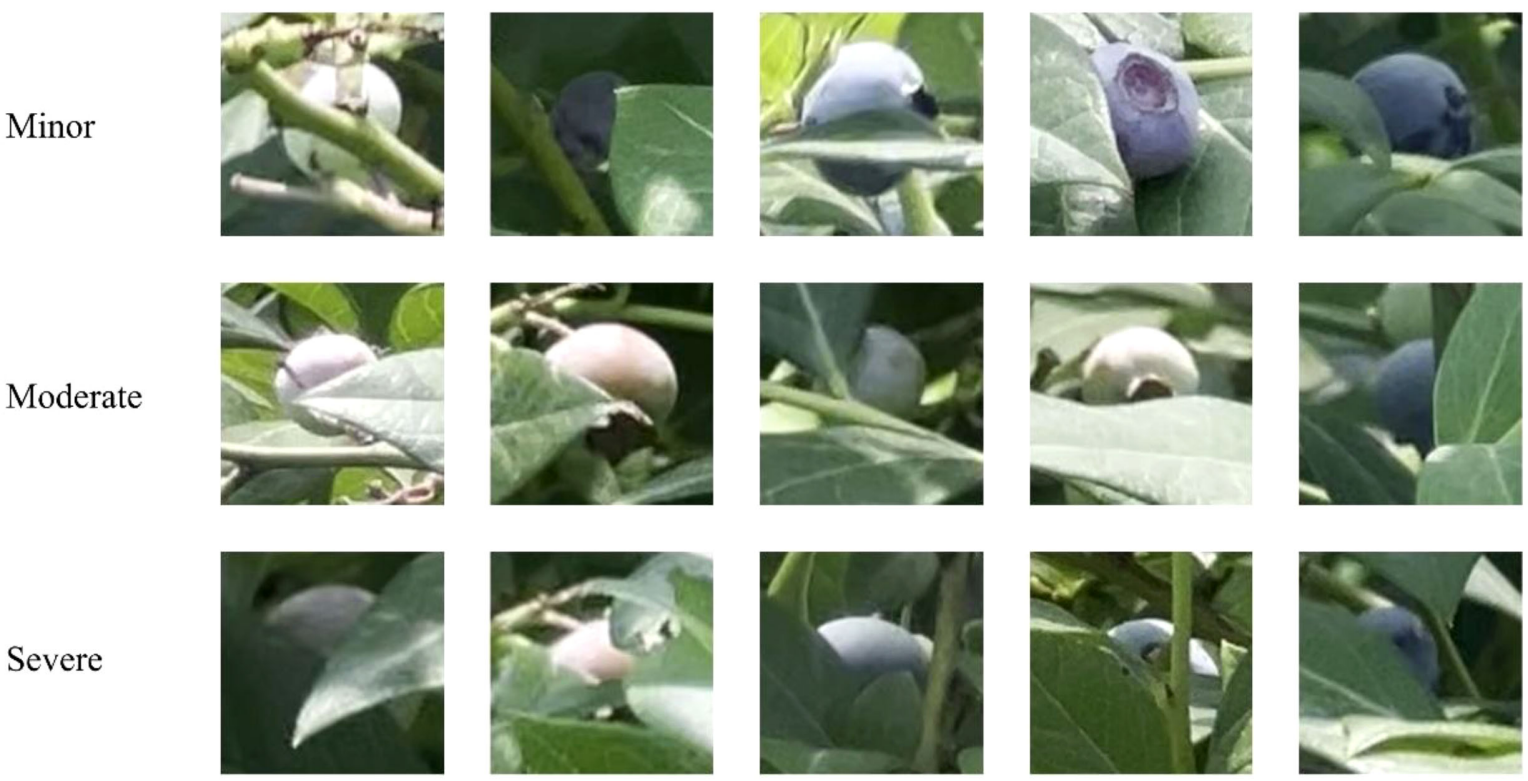
Visual classification criteria for occlusion challenges.

Second, severe occlusions remain a primary challenge leading to detection failures and counting errors. To quantify this issue, we categorized instances in the test set based on actual blueberry occlusion area into three levels: Minor (occlusion area < 50%), Moderate (occlusion area 50%–70%), and Severe (occlusion area > 70%). Visual examples are shown in **Figure 21**.

As shown in **Table 8**, which provides a direct comparison against the YOLOv8 baseline, STF-YOLO's superiority in handling occlusions is evident across all categories. Under Minor obstruction, STF-YOLO achieved a pass rate of 82.35%, representing a significant improvement over YOLOv8's 63.72%. This performance gap was maintained under Moderate obstruction, where our model achieved a 56.25% pass rate compared to the baseline's 46.87%.

**Table 8:** Obstruction pass rate.

Number of occlusion instances in the test set	Model	Degree	Numbers	Number of successful detections	Success rate
197	YOLOv8	Minor	102	65	63.72%
		Moderate	64	30	46.87%
		Severe	31	7	22.58%
	STF-YOLO	Minor	102	84	82.35%
		Moderate	64	36	56.25%
		Severe	31	10	32.26%

While performance for both models degrades substantially under Severe obstruction (e.g., the blurred boundary case shown in **Figure 20b**), STF-YOLO (32.26%) still outperformed YOLOv8 (22.58%) by a notable margin. This indicates that while our model shows marked improvements, its feature representation capability for heavily occluded targets remains a significant challenge. Therefore, future research should continue enhancing the model's feature representation ability, particularly its generalization and robustness in these extremely complex environments.

Additionally, deploying STF-YOLO in diverse agricultural settings presents several challenges. First, variable field conditions—such as fluctuating light levels and weather variations—can degrade detection accuracy. Second, the model's robustness across different crop varieties and growth stages must be thoroughly evaluated and optimized. Finally, to enable real-time system integration, STF-YOLO requires resource-aware optimization and calibration to balance cost constraints, hardware compatibility, and processing speed.

## 5 Conclusion

Accurate detection of blueberry maturity and precise fruit counting are essential for optimizing harvesting efficiency and improving economic returns in modern agriculture. In this study, a comprehensive blueberry video dataset was developed to capture the challenges of maturity evaluation and counting in real orchard environments. Leveraging this dataset, we developed a lightweight and efficient detection model, STF-YOLO. This model integrates four specialized modules—DSAA, AEF, MNS, and SDCH—to address key issues in maturity assessment, fruit occlusion, small-object detection, and model lightweight design. Experimental results demonstrate that STF-YOLO exhibits superior performance in detecting blueberries across all maturity stages. Compared with the original YOLOv8 model, STF-YOLO achieves improvements of 3.5% in both recall and mean average precision (mAP), while reducing computational complexity by 17.28%. Furthermore, STF-YOLO demonstrates superior performance across precision, recall, and efficiency metrics compared to other prevalent lightweight object detection models.

To evaluate generalization capability, STF-YOLO was tested on two distinct datasets: the agricultural-focused MegaFruit dataset and the diverse PASCAL VOC2007 dataset. On MegaFruit, STF-YOLO outperformed other YOLO variants, achieving the highest mAP scores for peach, strawberry, and blueberry categories. On VOC2007, STF-YOLO demonstrated state-of-the-art performance, confirming its robustness and adaptability across diverse object categories and complex real-world scenarios. Integrating STF-YOLO with the ByteTrack algorithm enabled automated blueberry counting in video sequences, achieving a counting accuracy of 72.49%. These results validate the reliability and practical applicability of STF-YOLO for real-world agricultural monitoring and automated harvesting systems. In summary, the proposed method provides an innovative solution for small-object fruit detection in complex agricultural scenarios. Future work will focus on expanding STF-YOLO's applicability to diverse crop types to enhance robustness and efficiency, thereby advancing intelligent and precision agriculture technologies.

## Data Availability

The raw data supporting the conclusions of this article will be made available by the authors, without undue reservation.
