# Supplementary figures and images for: Maturity detection and counting of blueberries in real orchards using a novel STF-YOLO model integrated with ByteTrack algorithm
Source: Front Plant Sci. 2025 Nov 27;16:1682024. doi: 10.3389/fpls.2025.1682024 (PMC12695768; doi:10.3389/fpls.2025.1682024)

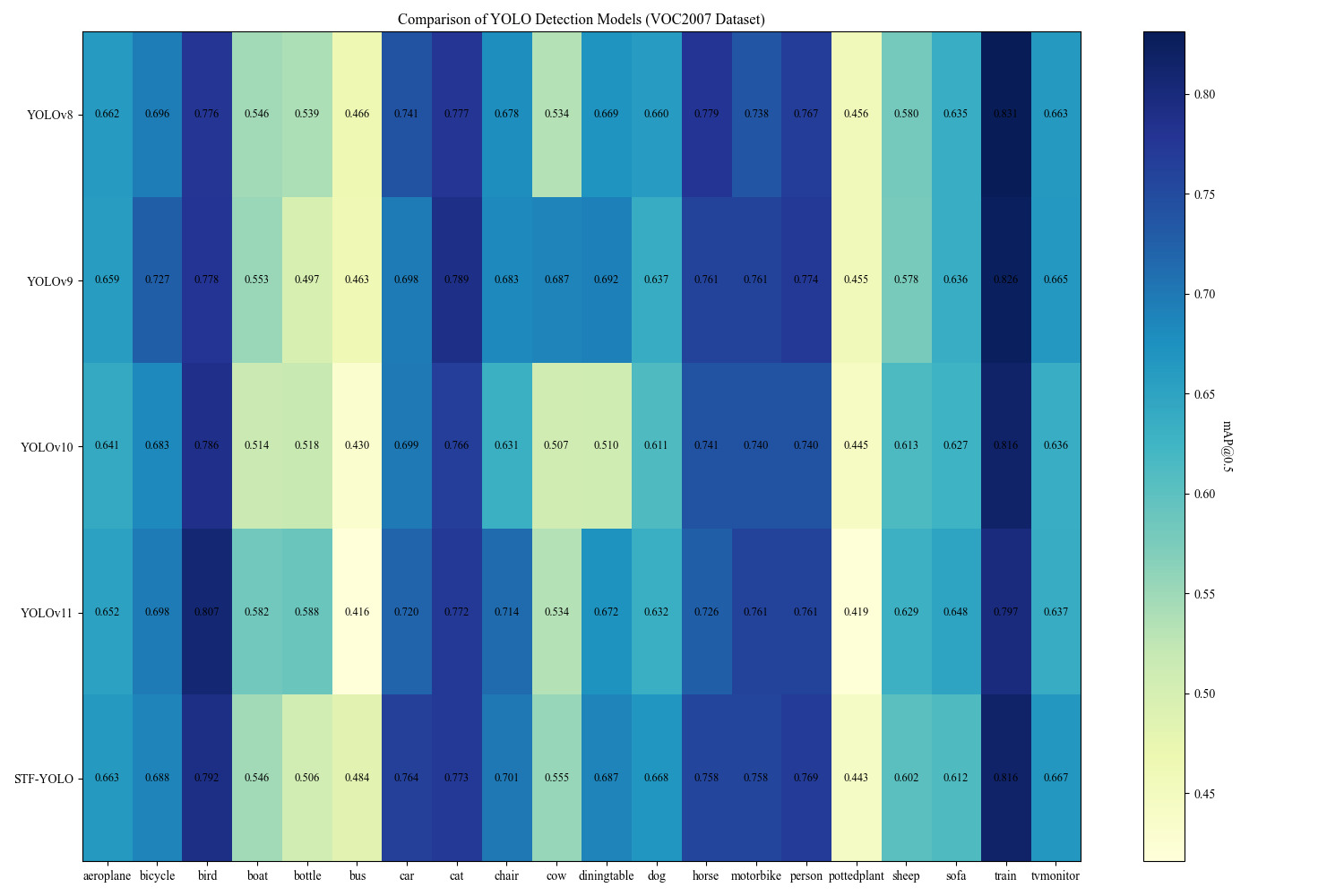

Supplement: Supplementary file 1 [file Image1.jpeg]

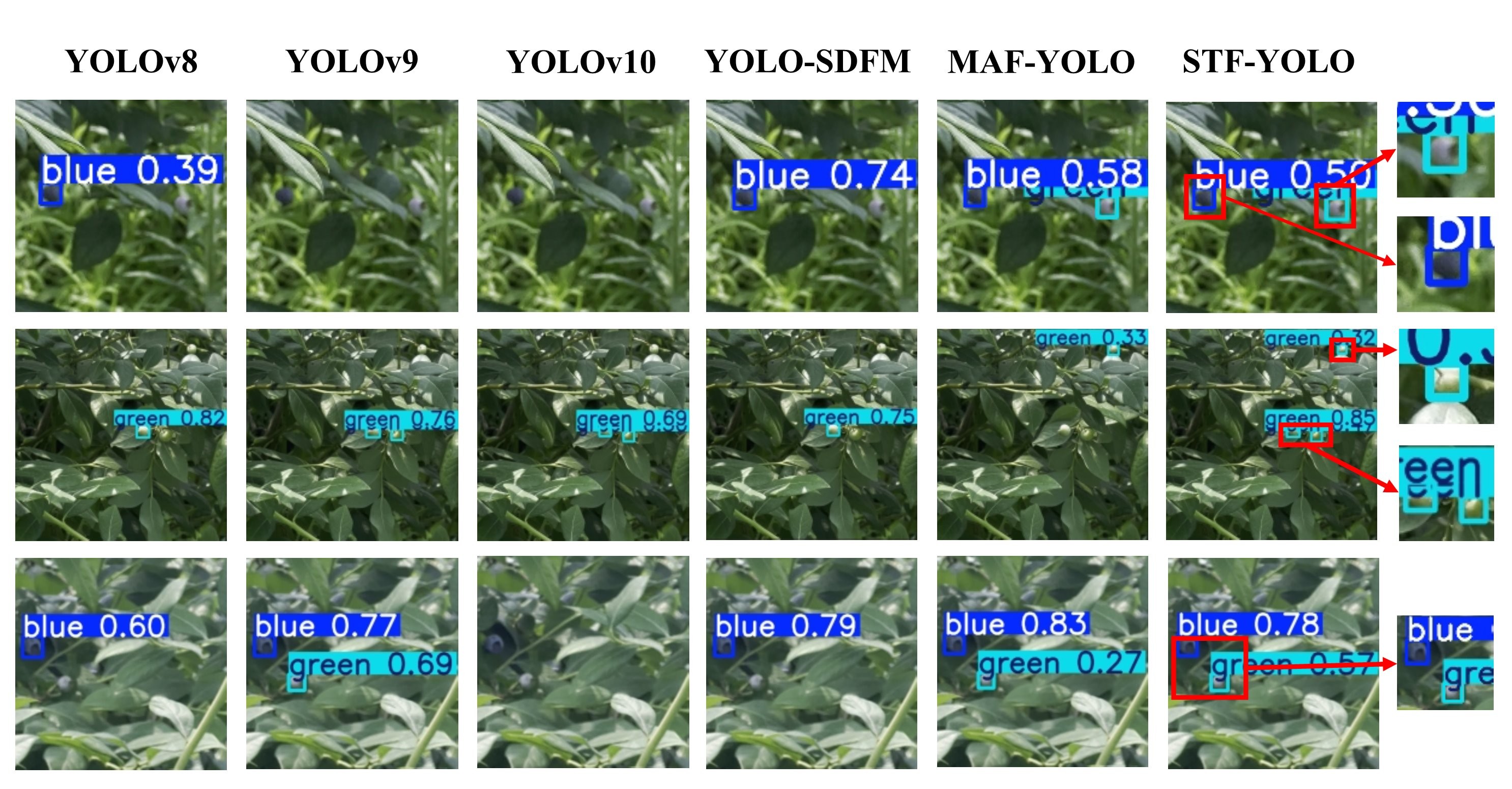

Supplement: Supplementary file 2 [file Image2.jpeg]

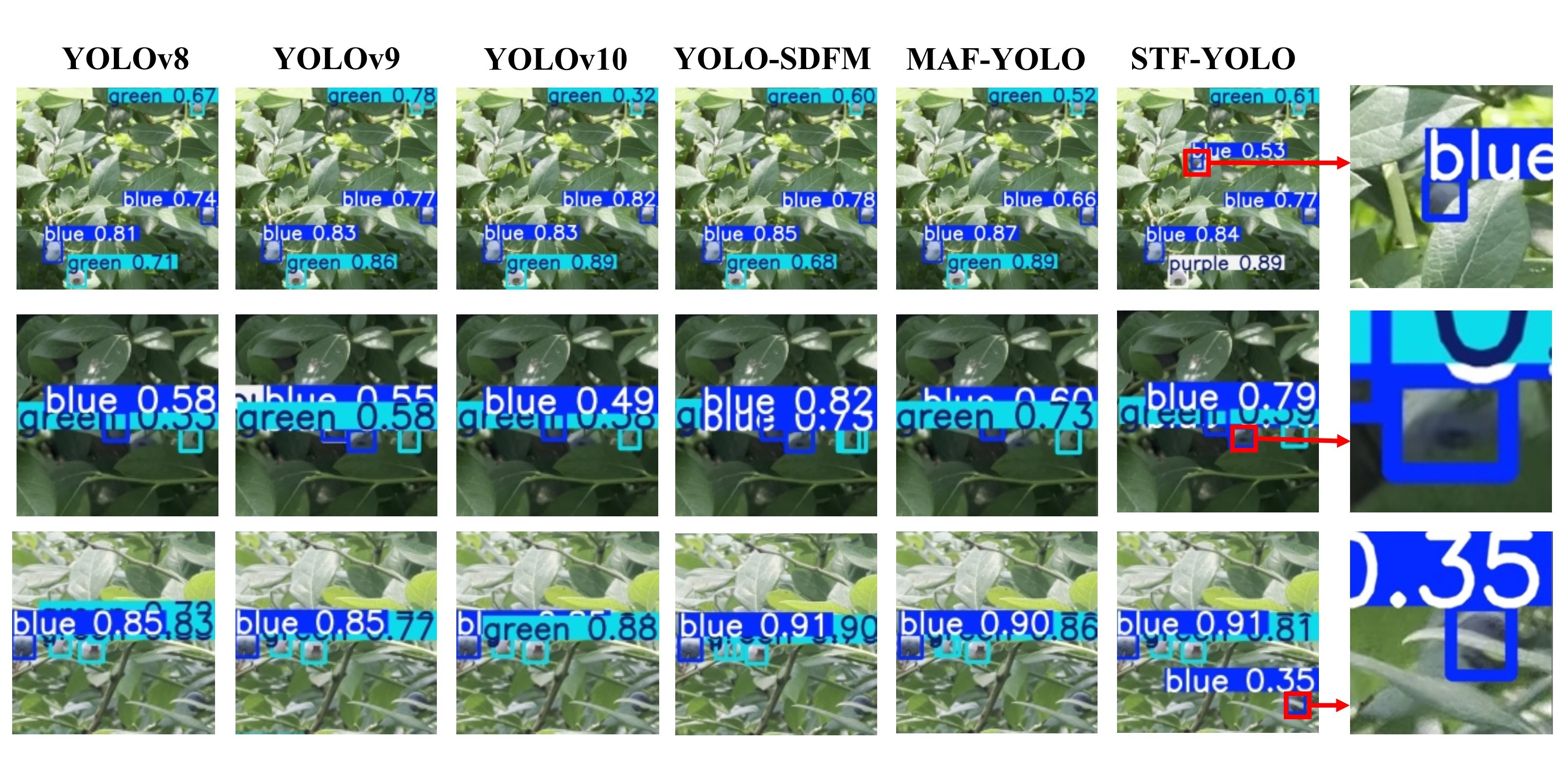

Supplement: Supplementary file 3 [file Image3.jpeg]

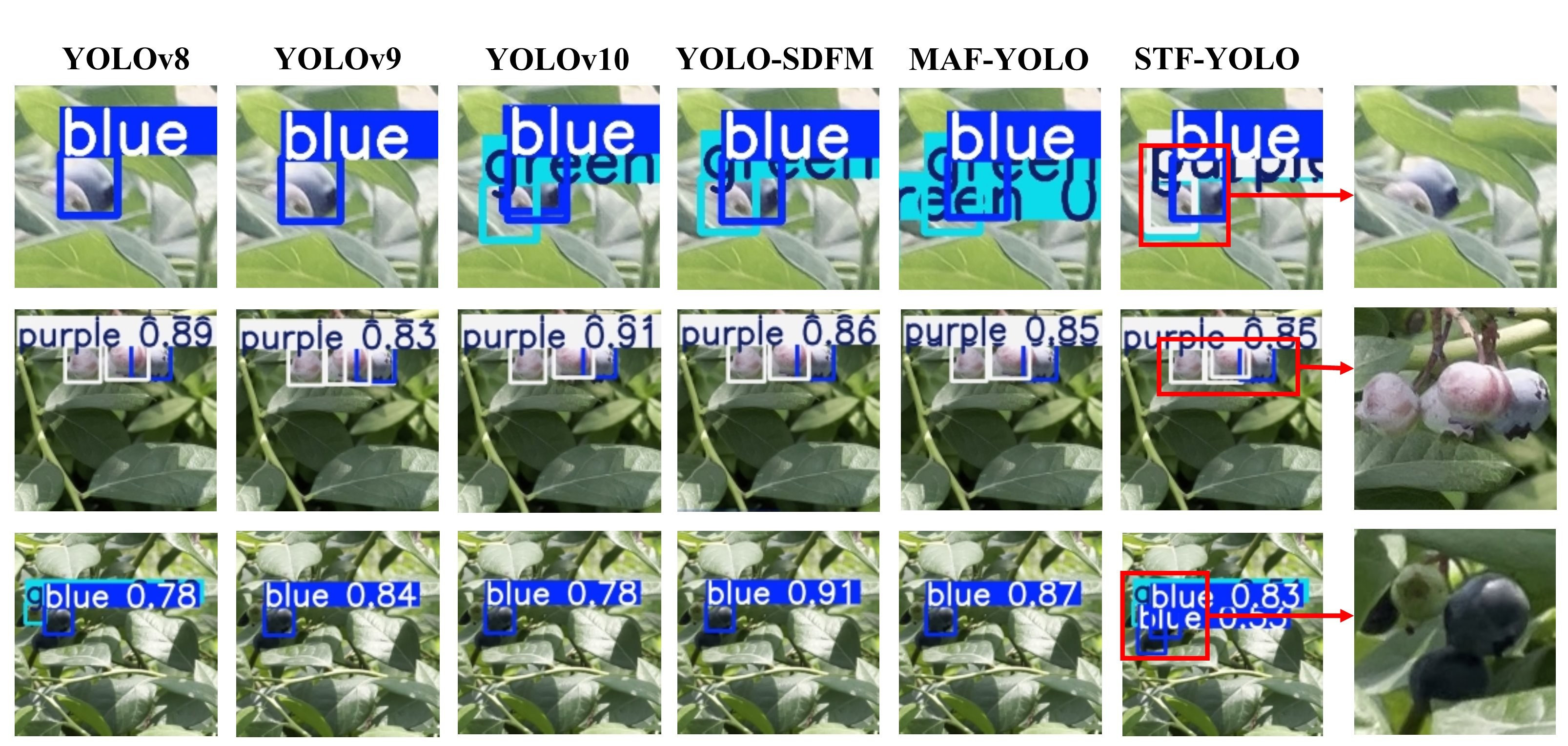

Supplement: Supplementary file 4 [file Image4.jpeg]

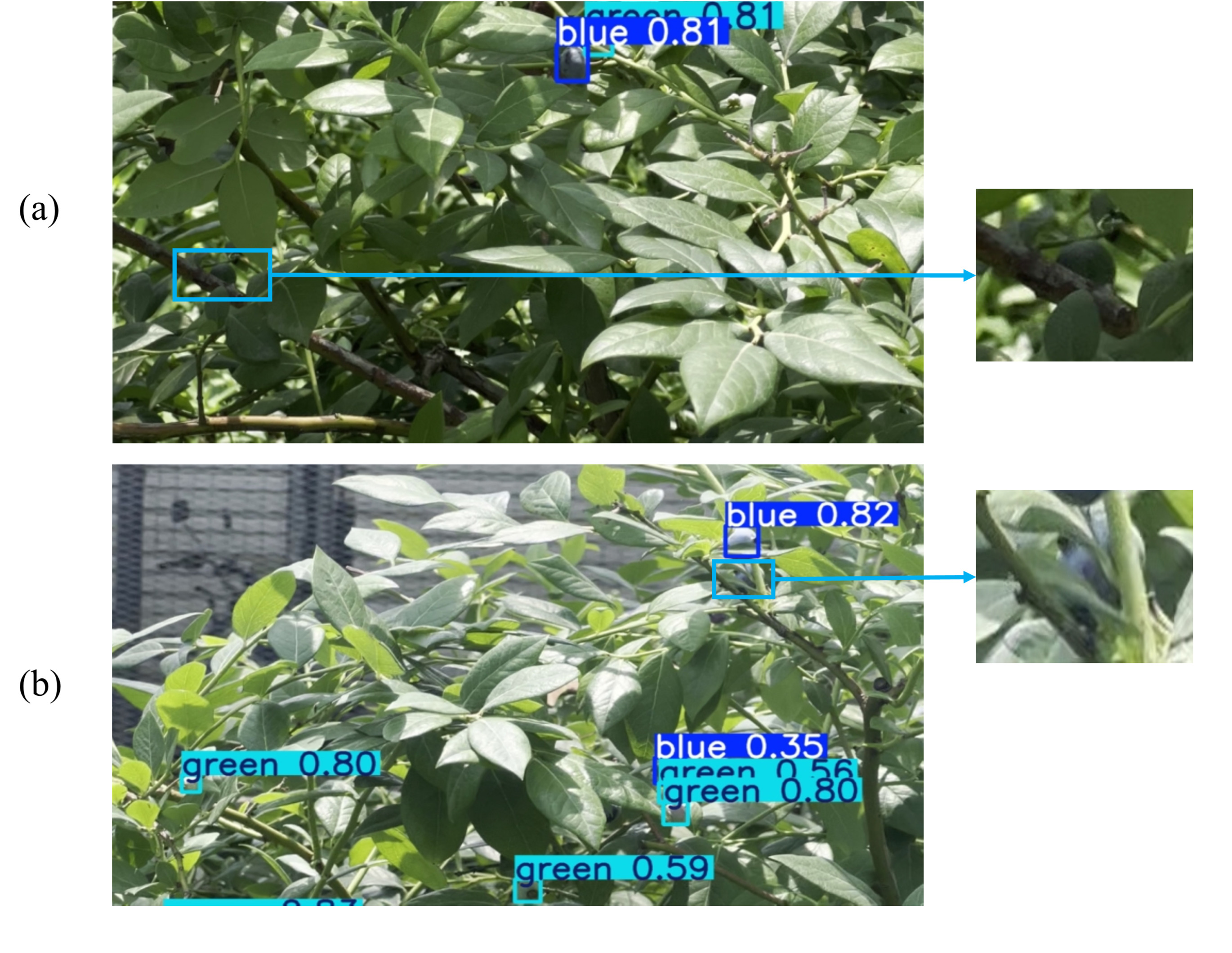

Supplement: Supplementary file 5 [file Image5.jpeg]

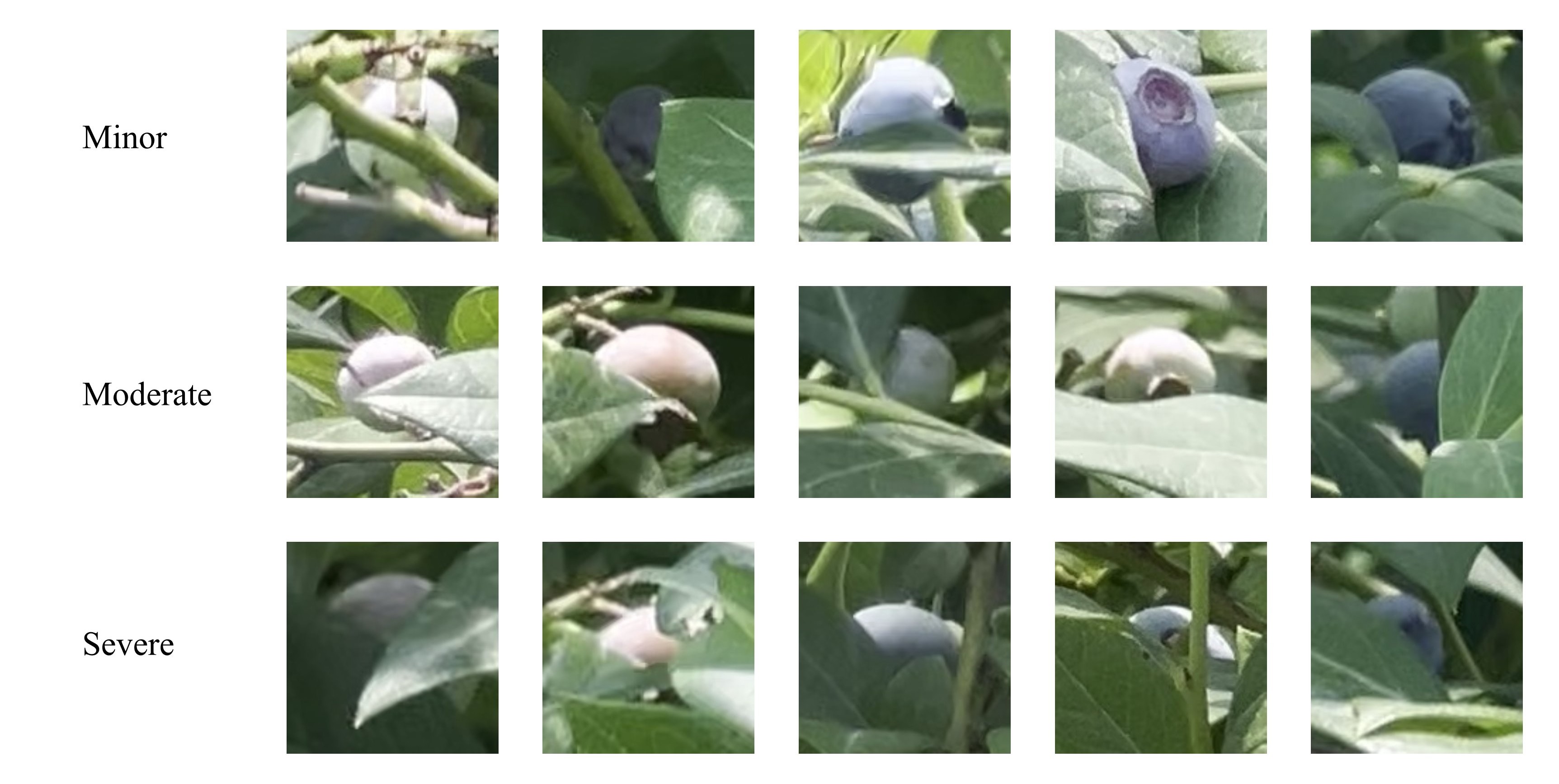

Supplement: Supplementary file 6 [file Image6.jpeg]

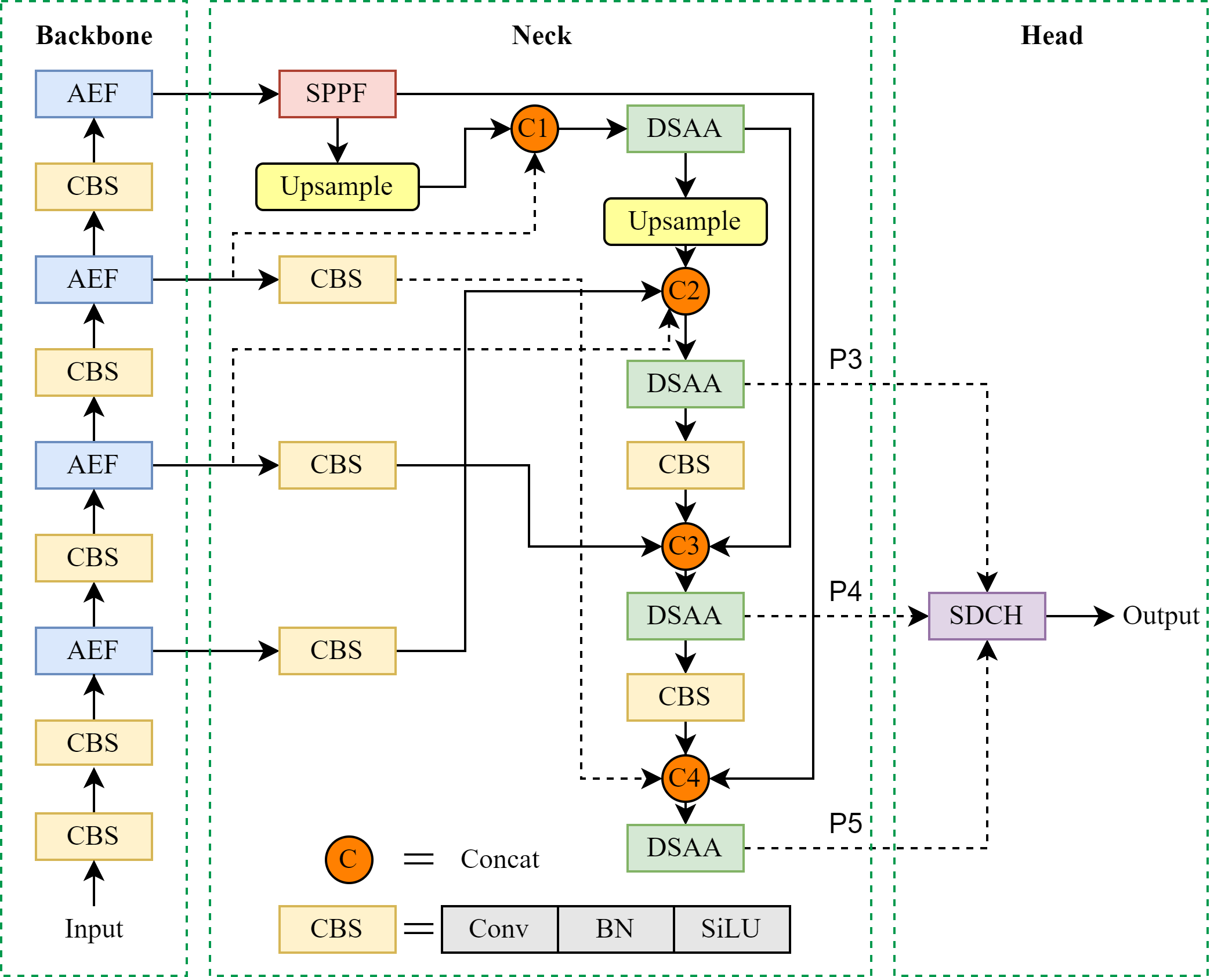

Supplement: Supplementary file 7 [file Image7.png]

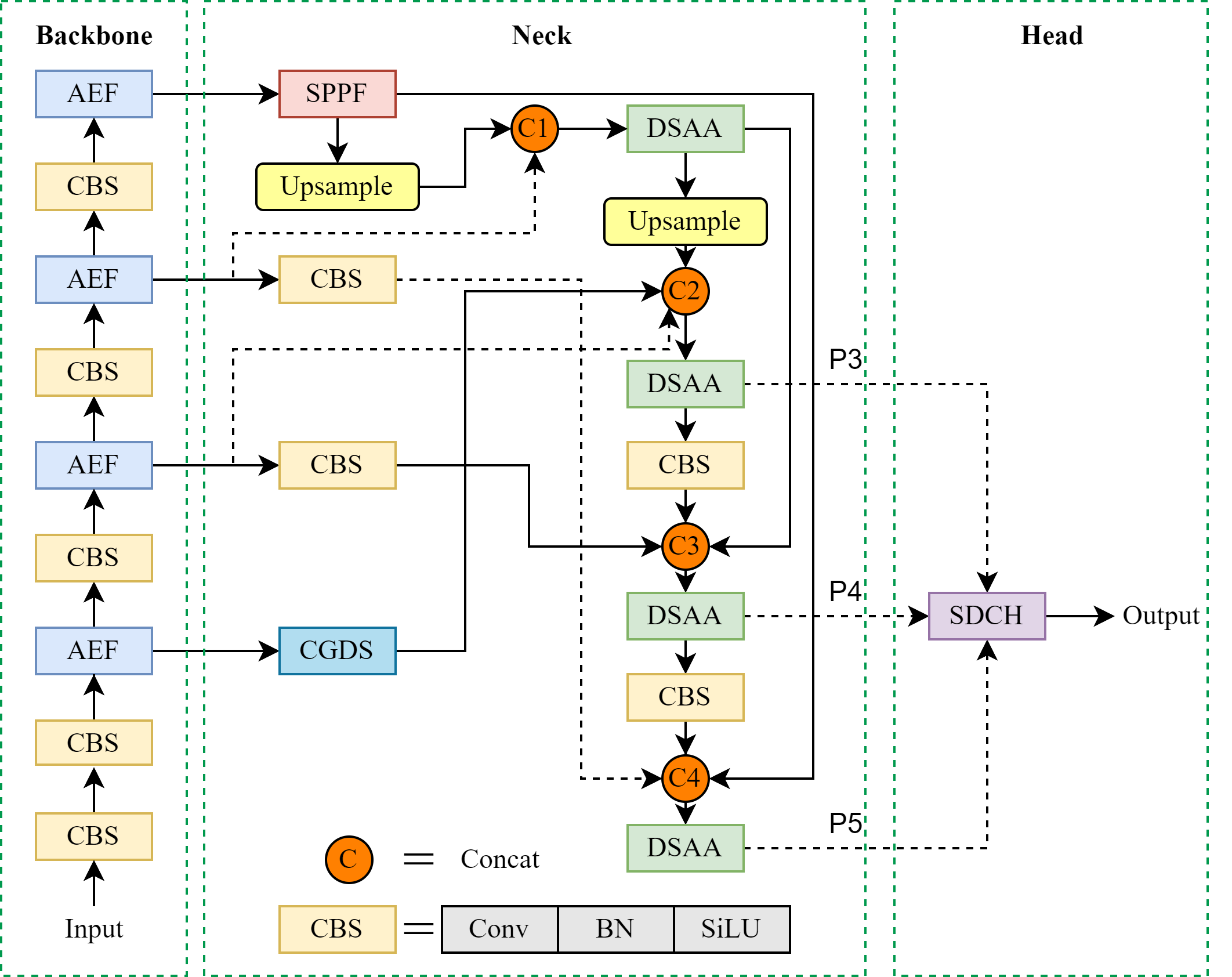

Supplement: Supplementary file 8 [file Image8.png]

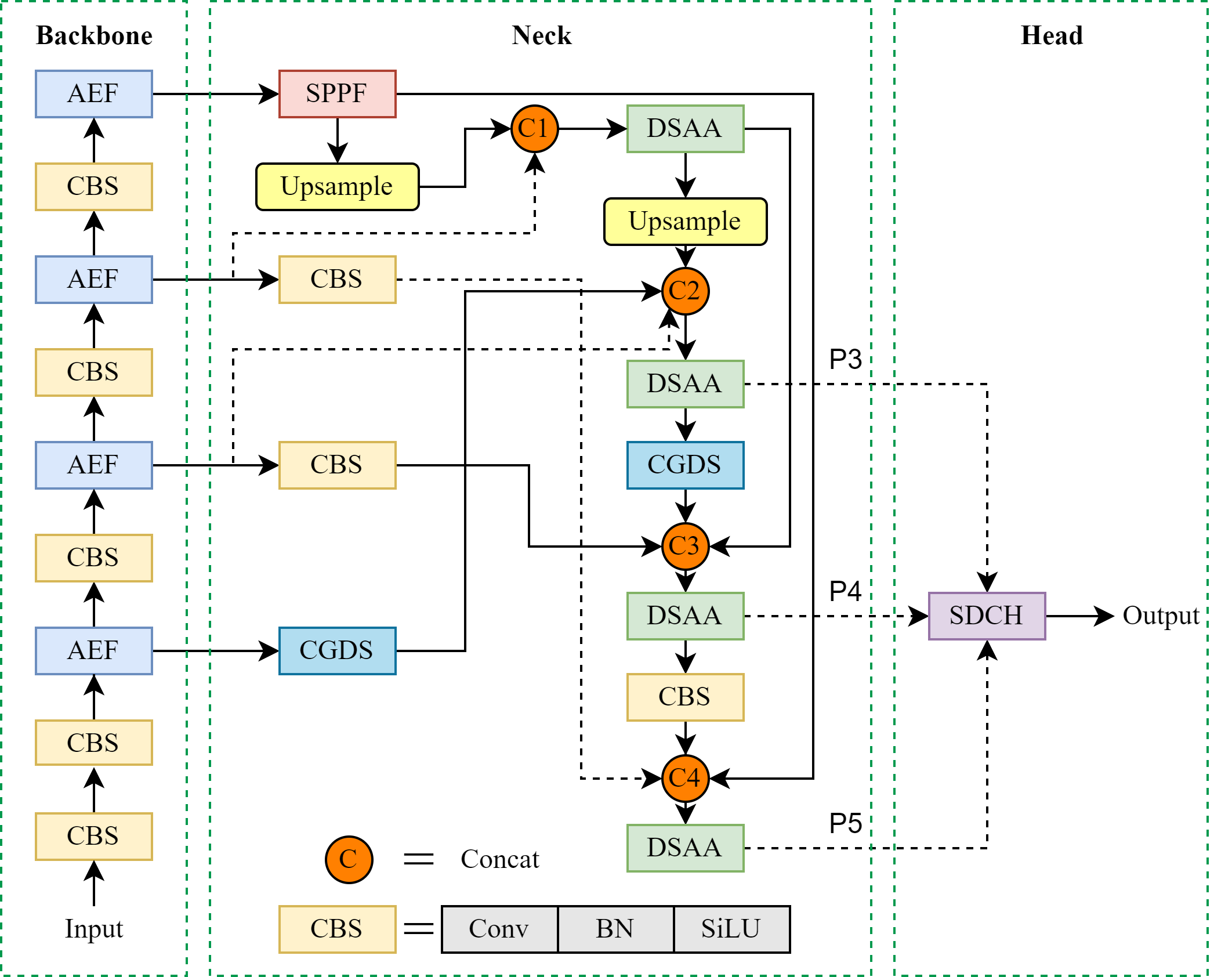

Supplement: Supplementary file 9 [file Image9.png]

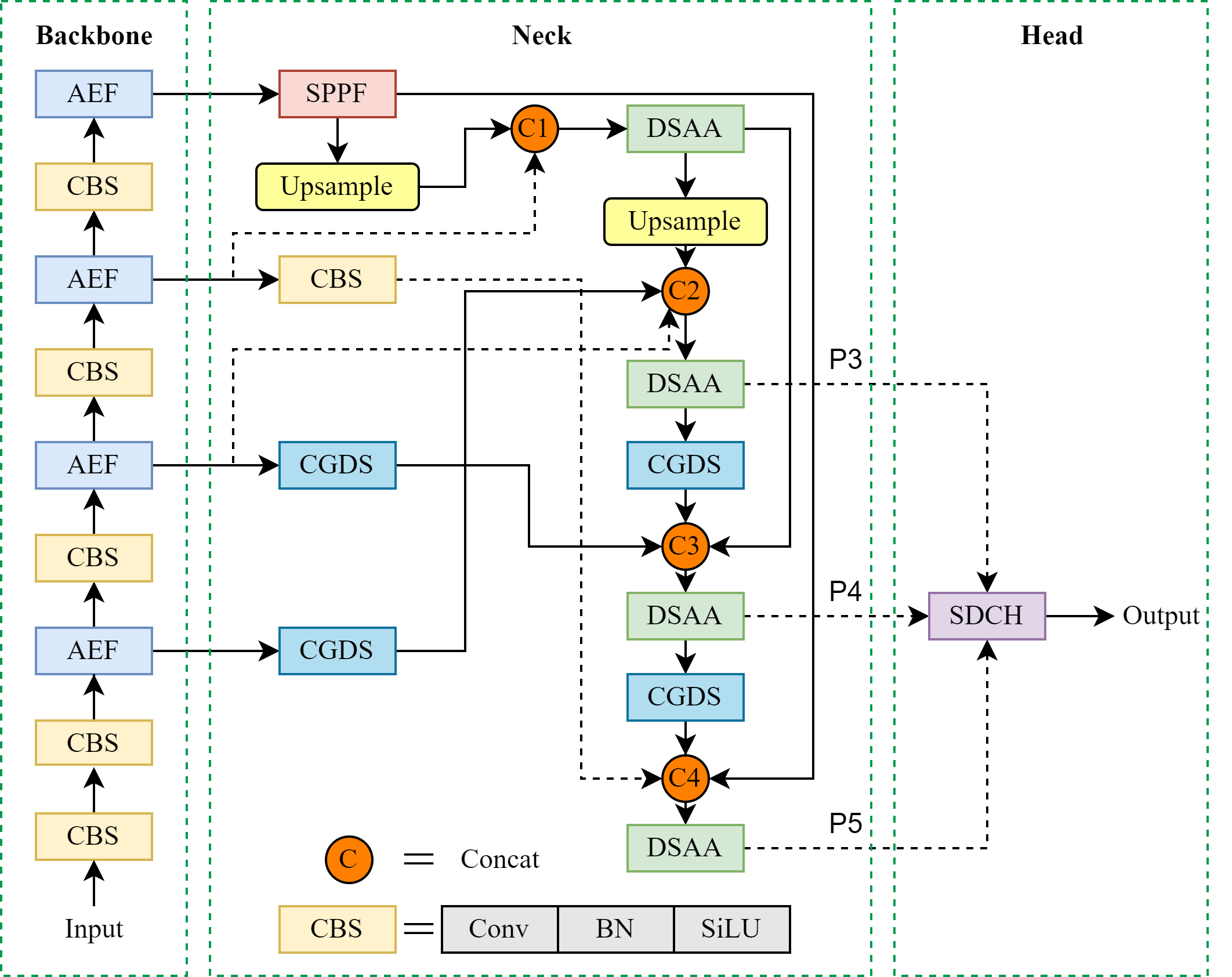

Supplement: Supplementary file 10 [file Image10.png]

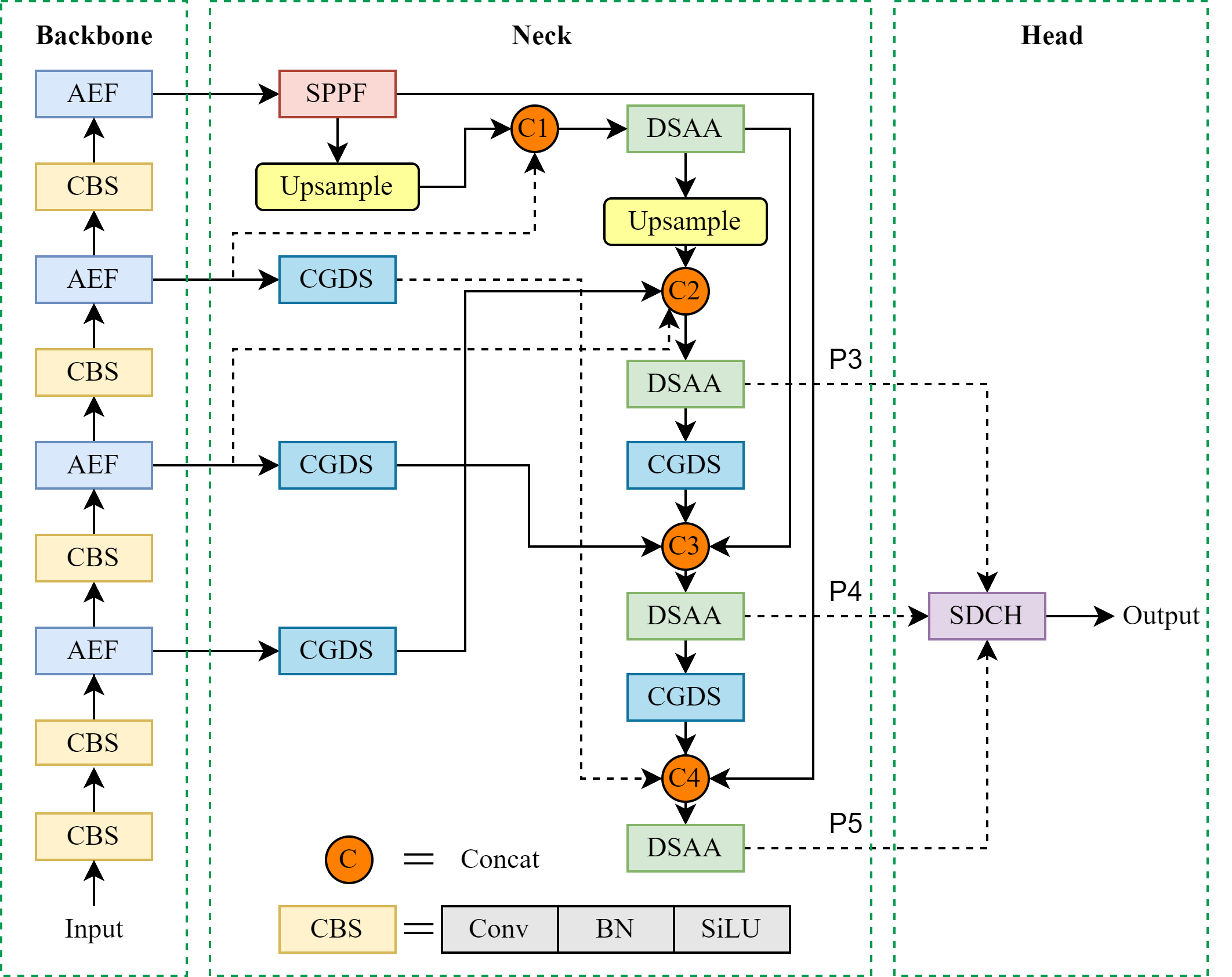

Supplement: Supplementary file 11 [file Image11.png]

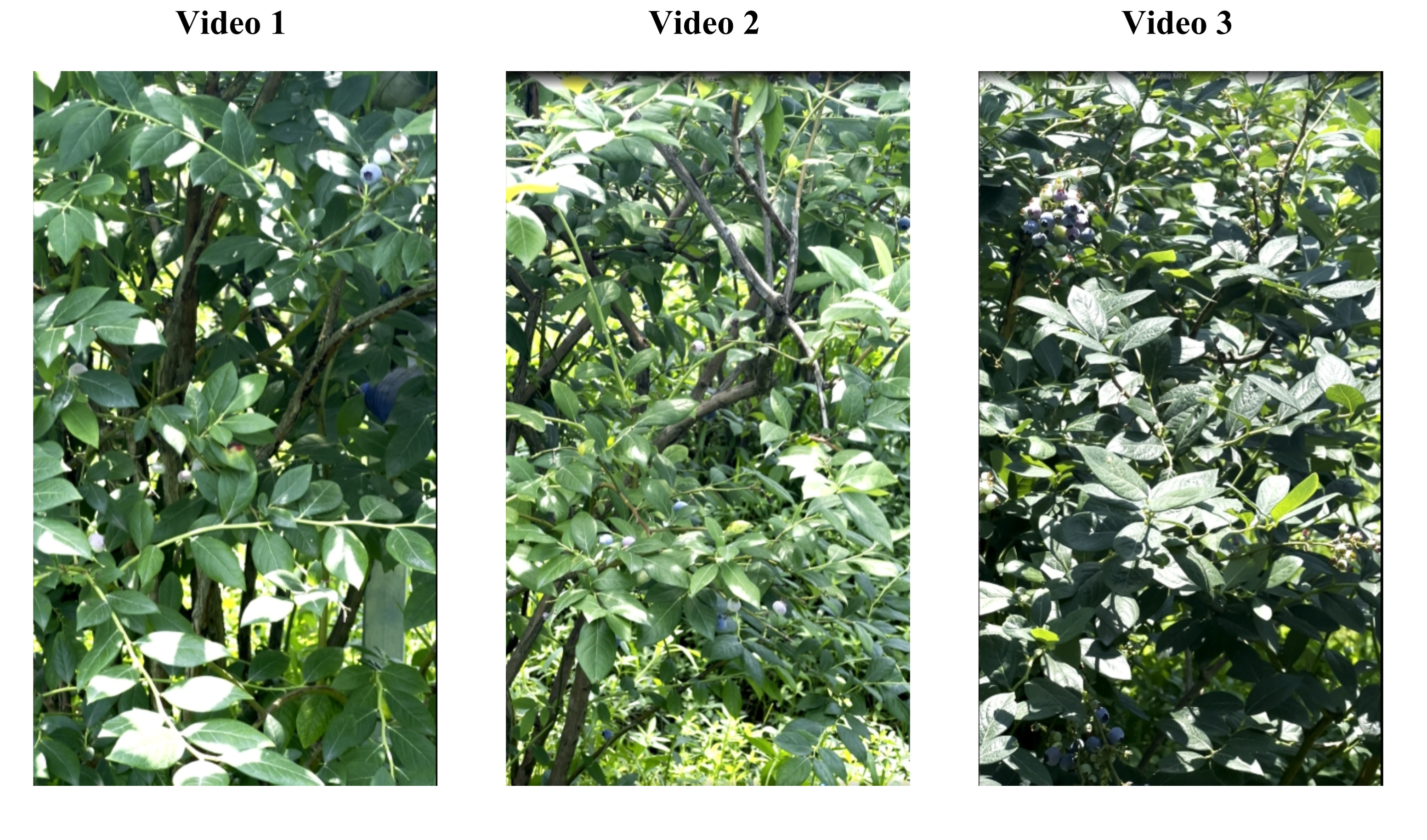

Supplement: Supplementary file 12 [file Image12.jpeg]
